# Facile conversion of ammonia to a nitride in a rhenium system that cleaves dinitrogen†

**DOI:** 10.1039/d1sc04503b

**Published:** 2022-03-04

**Authors:** Gannon P. Connor, Daniel Delony, Jeremy E. Weber, Brandon Q. Mercado, Julia B. Curley, Sven Schneider, James M. Mayer, Patrick L. Holland

**Affiliations:** Department of Chemistry, Yale University New Haven Connecticut USA patrick.holland@yale.edu; Institute of Inorganic Chemistry, Georg-August-Universität Göttingen Göttingen Germany

## Abstract

Rhenium complexes with aliphatic PNP pincer ligands have been shown to be capable of reductive N_2_ splitting to nitride complexes. However, the conversion of the resulting nitride to ammonia has not been observed. Here, the thermodynamics and mechanism of the hypothetical N–H bond forming steps are evaluated through the reverse reaction, conversion of ammonia to the nitride complex. Depending on the conditions, treatment of a rhenium(iii) precursor with ammonia gives either a bis(amine) complex [(PNP)Re(NH_2_)_2_Cl]^+^, or results in dehydrohalogenation to the rhenium(iii) amido complex, (PNP)Re(NH_2_)Cl. The N–H hydrogen atoms in this amido complex can be abstracted by PCET reagents which implies that they are quite weak. Calorimetric measurements show that the average bond dissociation enthalpy of the two amido N–H bonds is 57 kcal mol^−1^, while DFT computations indicate a substantially weaker N–H bond of the putative rhenium(iv)-imide intermediate (BDE = 38 kcal mol^−1^). Our analysis demonstrates that addition of the first H atom to the nitride complex is a thermochemical bottleneck for NH_3_ generation.

## Abstract 2

Rhenium–PNP complexes split N_2_ to nitrides, but the nitrides do not give ammonia. Here, the thermodynamics of the hypothetical N–H bond forming steps are evaluated through the reverse reaction, showing that the first H addition is the bottleneck.

## Graphical abstract

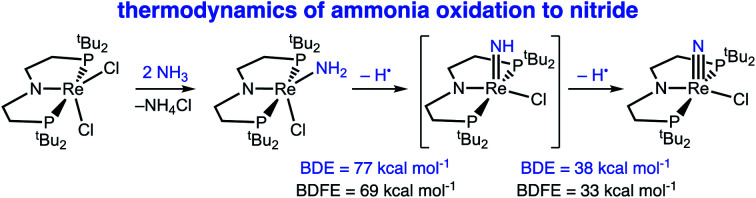

## Introduction

The interconversion of N_2_ and NH_3_ is important in fields that range from agriculture to sustainable energy.^1,2^ The heavy use of NH_3_ in fertilizer manufacturing has resulted in an extensive global infrastructure for its transportation and storage.^3^ Coupled with its high energy density, this makes NH_3_ an excellent candidate for a carbon-free chemical fuel, either through combustion or direct ammonia fuel cells (DAFCs).^4–7^ In order to realize this potential, it is beneficial to understand the individual steps of N–N and N–H bond formation and cleavage. One promising route to form the N–H bonds in NH_3_ from N_2_ is proton-coupled electron transfer (PCET).^8*a*,9^ Photo- or electrochemical energy may provide the necessary driving force for PCET-assisted N_2_ reduction using water as a source of protons and electrons, thus providing a sustainable strategy for converting N_2_ to NH_3_.^10–16^ A growing number of homogeneous systems catalytically achieve this difficult transformation utilizing PCET.^17–31^

It is also important to understand the reverse reaction, NH_3_ oxidation to form N_2_. One application of this reaction is for releasing the chemical energy stored in N–H bonds for DAFC applications.^6,7^ In addition, the individual steps in NH_3_ oxidation to N_2_ are often the microscopic reverse of those used for PCET reduction of N_2_ and thus help to elucidate potential mechanisms for PCET-assisted reduction of N_2_ to NH_3_.^32^ In this context, it is relevant that many examples of chemical N–H bond oxidation from NH_3_-derived metal ammines yield metal nitride complexes.^33–46^ These systems utilize either chemical oxidants under basic conditions or H-atom abstracting (HAA) reagents for the ammine-to-nitride transformations.^8*b*^ In some systems, electrochemical oxidation of ammine complexes yields metal–nitride products.^41,42,44^ Other systems can generate N_2_ as a product from the oxidation of NH_3_-derived ammine complexes, either through chemical^36,40,47–49^ or electrochemical^49–52^ methods. These include recently reported homogeneous systems that catalytically form N_2_ from NH_3_ through both chemical^43–45^ or electrochemical^44,53–55^ N–H bond oxidation. N–N bond formation can occur *via* bimetallic N–N coupling (*e.g.*, between metal–NH_*x*_ species or metal nitrides)^40,44,45^ or nucleophilic attack on a metal–NH_*x*_ intermediate by NH_3_.^43,53,54,56^

**Scheme 1:**
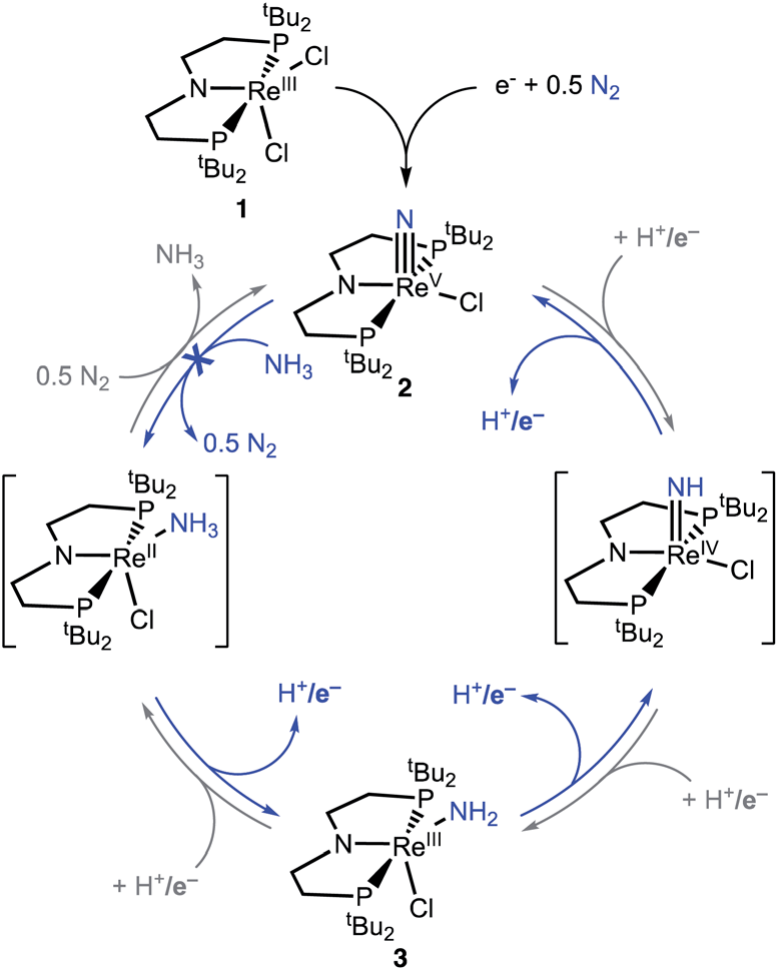
Cycles that represent reductive N_2_ splitting by (PNP)Re and PCET nitride reduction (gray cycle, not observed) or NH_3_ oxidation (blue cycle, studied here).

Here, we study NH_3_ oxidation in a well-defined system that is also capable of reductive functionalization of N_2_*via* an N_2_-cleavage mechanism.^57,58^ Electrochemical reduction of (PNP)ReCl_2_ (1, PNP = N((CH_2_CH_2_)P^t^Bu_2_)_2_) cleaves N_2_ to form the nitride complex (PNP)Re(N)Cl (2), which contains a nucleophilic nitride ligand (Scheme 1, black arrow).^59–61^ This nitride can be alkylated and reduced to give N–C containing products,^62,63^ but PCET reduction of the nitride in 2 to form NH_3_ (Scheme 1, grey arrows) was not observed because pincer protonation occurs rather than nitride protonation. Additional challenges are that the high energy of the lowest unoccupied molecular orbital (LUMO) of 2 prevents a reduction-first pathway, and that 2 is unreactive towards organic hydrogen-atom transfer (HAT) reagents or H_2_.^59^ In this manuscript, we evaluate the reverse reactions (Scheme 1, blue arrows) to elucidate the factors that prevent PCET nitride reduction in this system. This fundamental information may help to improve NH_3_ oxidation catalysis and to avoid bottlenecks in NH_3_ generation by future N_2_-cleaving systems, and importantly provides a thermochemical framework for nitrogen fixation products beyond ammonia.

## Results

### Binding and deprotonation of NH_3_

**Scheme 2:**
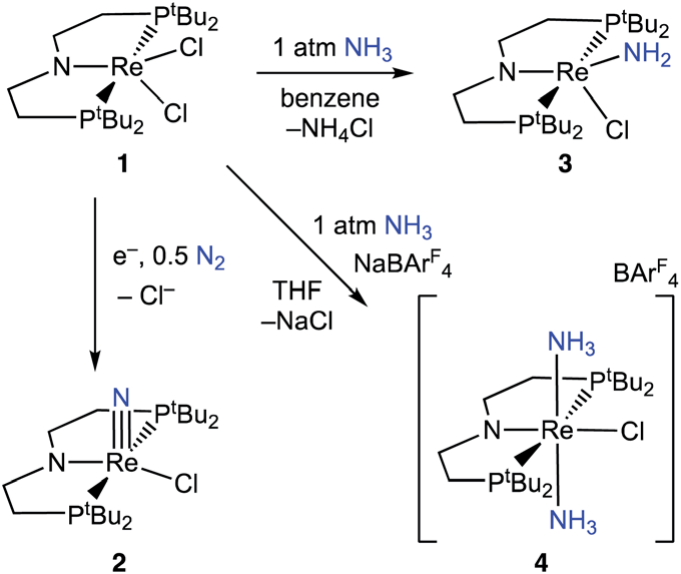
Reactivity of 1 with N_2_ and NH_3_.

Introduction of 1 atm of NH_3_ gas to a solution of the dichloride complex 1 in benzene-*d*_6_ or tetrahydrofuran-*d*_8_ (THF-*d*_8_) results in an immediate color change from purple to brown. ^1^H NMR spectroscopy reveals the formation of a new *C*_s_-symmetric product 3 in >95% yield (Scheme 2, top). The chemical shifts of 3 (Fig. S1†) are characteristic of a non-magnetic ground state non-, and the lack of noticeable temperature-independent paramagnetism, which is often observed in Re^III^ complexes,^64,65^ suggests that the two strongly π-donating amide ligands sufficiently destabilize the spin triplet state to give a well-isolated singlet ground state. A notable ^1^H resonance integrating to 2H is found at *δ* 12.7 ppm. A ^1^H–^15^N HSQC spectrum of a natural-abundance sample shows a ^15^N cross-peak from this resonance at *δ* –260 ppm (Fig. S2†), confirming that it corresponds to protons bound to N. This ^15^N chemical shift is significantly upfield from related nitride complexes (371–393 ppm) and closer to that for the protonated P*N*P backbone of [(^H^PNP)Re(N)Cl]^+^ (5) (−336 ppm).^66^ All spectroscopic signatures are consistent with the formulation of 3 as the amido complex (PNP)Re(NH_2_)Cl.

**Fig. 1:**
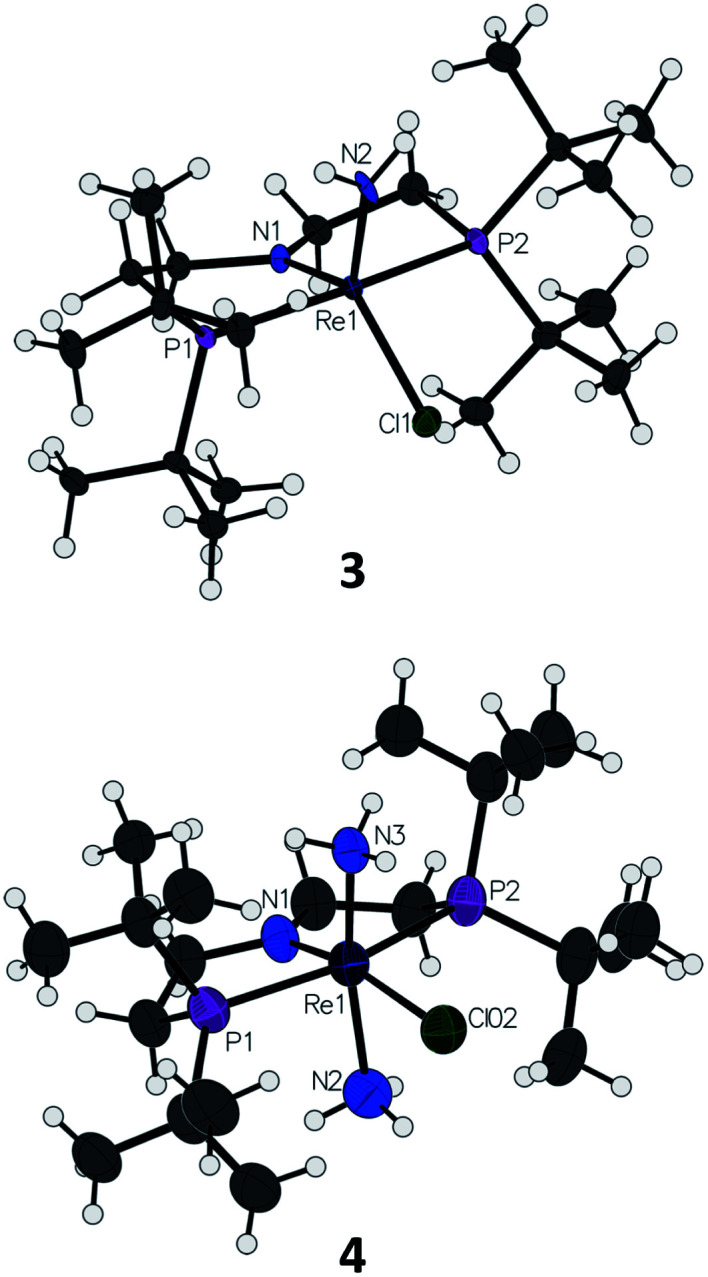
Solid-state structures of Re–amide complex 3 and Re(NH_3_)_2_ complex 4 (BAr^F^_4_ ion omitted) with thermal ellipsoids at 50% probability.

On a preparative scale, addition of 1 atm NH_3_ to a solution of 1 gives 3 as the major product, which is isolated from the reaction in 61% yield. The solid-state structure of 3 was elucidated *via* single crystal X-ray diffraction (XRD) and the N-bound hydrogen atoms were located in the Fourier map (Fig. 1). The Re–*N*H_2_ bond in 3 is 0.3 Å longer than the Re≡*N* bond in the nitride complex 2 (Table 1).^66^ In the supporting ligand, the (P*N*P)–Re bond is 0.1 Å shorter in 3 than in the Re–nitride complex 2, indicating increased π-bonding from the nitrogen of the pincer ligand in 3 (Table 1). The (P*N*P)–Re and Re–*N*H_2_ amide bond lengths in 3 are within 0.02 Å of each other with planar coordination of the nitrogen atoms in both cases (*Σ*_P*N*P_ = 360°, *Σ*_*N*H_2__ = 357°). The PNP and NH_2_ amides are oriented to π-donate into the same Re d orbital, which gives modest lengthening (0.04 Å) of the (P*N*P)–Re bond in 3 compared to the Re–dichloride complex 1.^59^ This also likely contributes to increased pyramidalization of the dialkylamide group (*Σ*_P*N*P_ = 348°) in 2. These structural differences are accompanied by a change of the rhenium coordination geometry from square pyramidal (*τ*_5_ = 0.14) in complex 2, in which the coordination site *trans* to the nitride is open, toward trigonal bipyramidal (*τ*_5_ = 0.48) in complex 3.

**Table 1:** Selected bond lengths (Å) and bond angles (°) of complexes 2–4

Bond/angle	2	3	4
Re1–N1	2.033(6)	1.936(3)	1.894(5)
Re1–N2	1.643(6)	1.959(3)	2.150(5)
Re1–N3	—	—	2.193(6)
Re1–Cl1	2.441(2)	2.384(1)	2.495(2)
Re1–P1	2.443(2)	2.397(1)	2.424(2)
Re1–P2	2.435(2)	2.382(1)	2.425(2)
N1–Re1–N2	105.8(3)	115.5(1)	84.8(2)
N1–Re1–N3	—	—	165.6(2)
N2–Re1–N3			109.6(2)
N1–Re1–Cl1	106.5(2)	108.8(1)	83.4(1)
N2–Re1–Cl1	147.7(2)	135.6(1)	167.0(2)
N1–Re1–P1	100.4(2)	95.1(1)	91.0(1)
N1–Re1–P2	99.9(2)	95.5(1)	90.5(1)

**Fig. 2:**
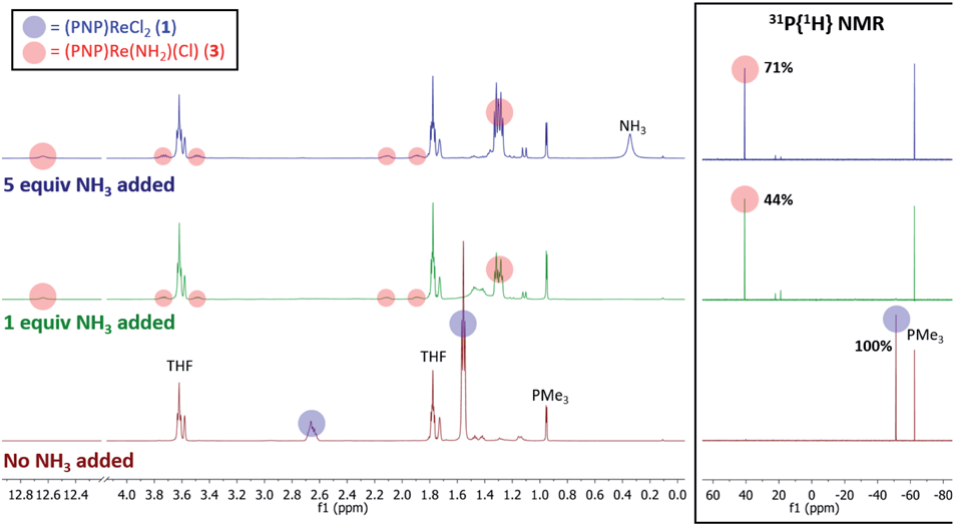
^1^H and ^31^P{^1^H} NMR spectra of 1 in THF-*d*_8_ without NH_3_ (bottom, maroon), with 1 equiv. NH_3_ added (middle, green), and with 5 equiv. NH_3_ added (top, blue). Spectroscopic yields reported *vs.* PMe_3_ in a capillary.

When 1 equiv. of NH_3_ gas was added to a solution of 1 in THF-*d*_8_ at −80 °C, ^1^H and ^31^P{^1^H} NMR spectra of the reaction showed a mixture of diamagnetic products (Fig. 2, middle). Addition of another 4 equiv. of NH_3_ gas (for a total of 5 equiv. NH_3_ per Re) resulted in full consumption of 1 and observation of 3 in 71% yield (Fig. 2, top). It is likely that dehydrohalogenation of the putative intermediate (PNP)Re(NH_3_)Cl_2_ by NH_3_ to form NH_4_Cl is required to drive the formation of 3. This implies that coordination to Re^III^ significantly increases the acidity of the N-bound protons.^67^

Addition of 1 atm NH_3_ to a solution of 1 containing an equivalent of NaBAr^F^_4_ (Ar^F^ = 3,5-bis(trifluoromethyl)phenyl) in THF-*d*_8_ at −80 °C resulted in a color change from purple to light green and formation of the new diamagnetic complex 4 by ^1^H and ^31^P{^1^H} NMR spectroscopy (see Scheme 2). In contrast to 3, complex 4 exhibits *C*_2v_ symmetry, broadened resonances, and a new peak at *δ* = 5.47 ppm that integrates to 6H (Fig. S3†). Despite no identifiable cross-peaks in the ^1^H–^15^N HSQC spectrum of 4, N–H stretching bands were observed in the infrared (IR) spectrum at 3392, 3353, 3245, and 3174 cm^−1^. The molecular structure of 4 in the solid state shows the six-coordinate, cationic bis-ammine adduct [(PNP)Re(NH_3_)_2_Cl][BAr^F^_4_] with a distorted octahedral geometry (Fig. 1). In comparison to 3, complex 4 shows lengthened Re–*N* bonds (2.172(5) Å *vs.* 1.959(3) Å) due to the lack of π-donation. With no strong π-donor ligands to compete with π-donation from the P*N*P amide, 4 contains a Re–P*N*P bond distance that is shorter than in 3 and 2 (Table 1). The flexibility of the P*N*P–Re interaction to accommodate the changes in ligand donor characteristics from ammine to nitride is also evident from the change in the P*N*P–Re bond lengths and P*N*P pyramidalization from 2–4.

### Reactivity of [(PNP)Re(NH_3_)_2_Cl]^+^

**Scheme 3:**
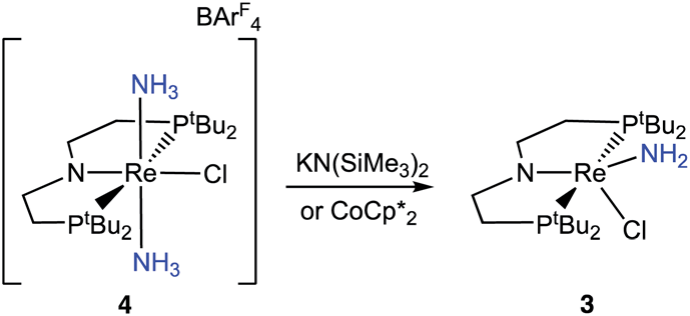
Reactivity of 4 with stoichiometric base or reductant.

To assess the plausibility of an ammine complex as an intermediate during formation of 3, a solution of 4 in THF-*d*_8_ was treated with 1 atm of NH_3_, which gave no reaction. However, addition of a slight excess of potassium hexamethyldisilazide (KHMDS) (Scheme 3) caused an immediate color change and formation of 3 as the major product in 61% yield, as judged by ^1^H and ^31^P{^1^H} spectroscopy (Fig. S4†).

Cyclic voltammetry (CV) of 4 in THF under Ar shows irreversible redox processes, a reduction at *E*_pc_ = −1.95 V *vs.* Cp_2_Fe^+/0^ and an oxidation at *E*_pa_ = −0.58 V *vs.* Cp_2_Fe^+/0^ (Fig. S16†). The position of the reduction peak is similar to the reversible reduction of 1 under Ar at −2.00 V *vs.* Cp_2_Fe^+/0^.^68^ The first reduction of 1 under Ar was previously attributed to the formation of [(PNP)Re^II^Cl_2_]^−^, which is followed by chloride dissociation to form (PNP)Re^II^Cl which is subsequently reduced again.^60^ The difficulty of reducing 4 suggests that it is quite electron-rich despite its positive charge, but the lack of reversibility prevents further interpretation.

In an attempt to assess the species formed upon reduction, 4 was treated with a chemical reductant. Addition of 1.2 equiv. CoCp*_2_ to a solution of 4 in THF-*d*_8_ under N_2_ gave complete consumption of 4 but the Re amide 3 was formed (Fig. S5†). The spectroscopic yield of 3 was only 60%. The fate of the lost proton and electron in the formation of 3 remain unknown. Analysis of the headspace following the reaction showed no detectable amount of H_2_ (<1% yield).

### N–H abstraction from Re–amide complex (PNP)Re(NH_2_)Cl

**Scheme 4:**
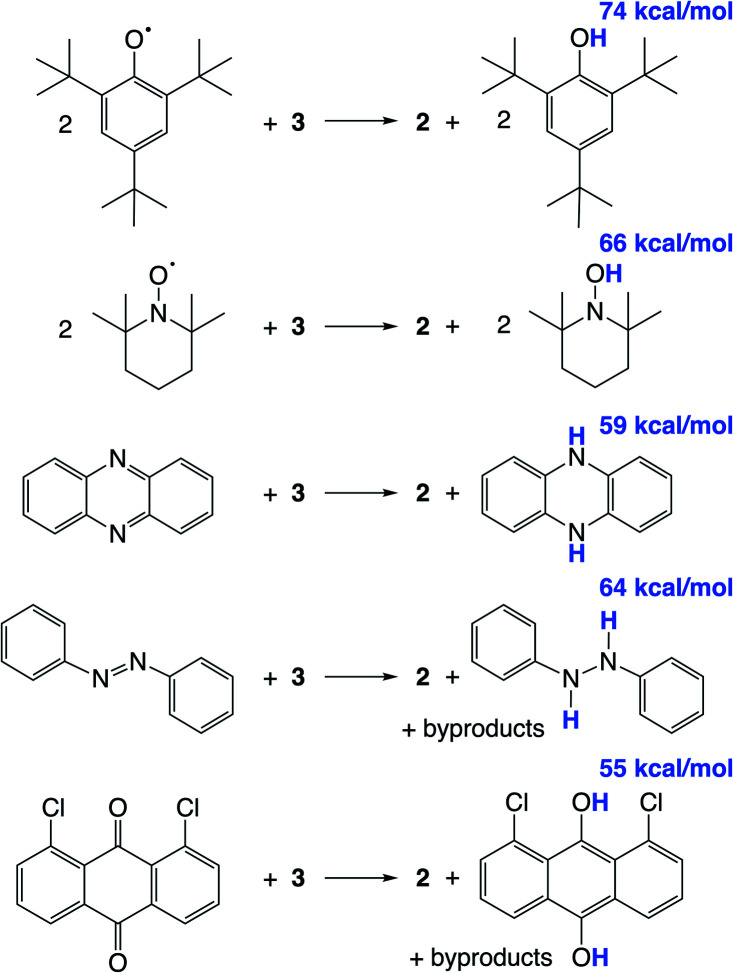
Reactivity of 3 with organic HAA reagents, with BDFE_O–H_ or average BDFE_X–H_ of the organic products in THF given in blue.

We hypothesized that abstraction of H atoms from 3 would lead to the nitride (Scheme 1, blue), by analogy with other reported systems.^37–40,43,45,46^ In the following, we assume that formal H˙ abstraction by the hydrogen atom abstraction (HAA) reagents is most likely concerted, based on the known difficulty of stepwise PCET pathways.^8^ Addition of 2 equiv. of either 2,4,6-tri-*tert*-butylphenoxyl radical (^*t*^Bu_3_PhO˙) or 2,2,6,6-tetramethylpiperidine 1-oxyl (TEMPO˙) as HAA reagents to a solution of 3 in THF-*d*_8_ or benzene-*d*_6_ at ambient temperature gives rapid and quantitative (>99%) formation of 2 (Scheme 4), as judged by ^1^H and ^31^P{^1^H} NMR spectroscopy (Fig. S6†). This is accompanied by the formation of 2 equiv. of ^*t*^Bu_3_PhOH or TEMPOH. These reagents have O–H bond dissociation free energies (BDFE_O–H_) of 74.4 and 65.5 kcal mol^−1^ in THF, respectively.^8*b*,69^ When 3 is mixed with only 1 equiv. of TEMPO˙, only half of 3 is consumed, showing that the second H-atom abstraction is more favorable than the first (Fig. S7†). The absence of reactivity of 2 with excess ^*t*^Bu_3_PhOH or TEMPOH supports this notion.

Additional HAA reagents were used to further bracket the N–H bond strengths (Scheme 4). While 3 did not react with 5,10-phenazine (5 equiv) in THF-*d*_8_ at ambient temperature, heating to 80 °C gave quantitative (>98%) conversion to 2 and 5,10-dihydrophenazine (average BDFE_N–H_ = 58.7 kcal mol^−1^ in MeCN^69^) after 21 h (Fig. S8†). Accordingly, no reaction was observed between 2 and 10 equiv. of 5,10-dihydrophenazine even after prolonged heating at 80 °C. Oxidation of 3 to form 2 was also observed when using 1.5 equiv. of azobenzene (54% yield of 2 after 72 h at 60 °C) and 1,8-dichloro-9,10-anthraquinone (67% yield of 2 after 4 d at ambient temperature). ^1^H NMR spectra of reaction mixtures showed the formation of 1,2-diphenylhydrazine and 1,8-dichloro-9,10-anthracenediol (average BDFE_X–H_ = 60.9 [in MeCN] and 55.4 kcal mol^−1^ [THF]), respectively.^69^ However, these reactions form multiple products, so quantitative thermochemical information cannot be derived from the product formation in these cases. Quantification of the PCET thermochemistry was therefore carried out by titration calorimetry as detailed below.

### Stepwise ET-PT from (PNP)Re(NH_2_)Cl

The reactivity of 3 with HAA reagents suggests that the N–H bonds in the amide ligand can be easily oxidized *via* concerted removal of an H-atom.^8^ We were also interested to determine whether the conversion to 2 is possible through stepwise PCET, with deprotonation and 1e^−^ oxidation of 3.^41,42,44^ In order to test a PT-ET (proton transfer followed by electron transfer) pathway, a solution of 3 was mixed with up to 12 equiv. of 1,8-diazabicyclo[5.4.0]undec-7-ene (DBU, p*K*_a_ of conjugate acid = 16.9), phosphazene base P_1_-tBu-tris(tetramethylene) (p*K*_a_ of conjugate acid = 20.2), or phosphazene base P_4_-tBu (p*K*_a_ of conjugate acid = 33.9) in THF-*d*_8_ at ambient temperature.^70^ No reaction of 3 with any of these strong bases was observed by ^1^H and ^31^P{^1^H} NMR spectroscopy, indicating that the amide ligand in 3 is a poor Brønsted acid.

**Fig. 3:**
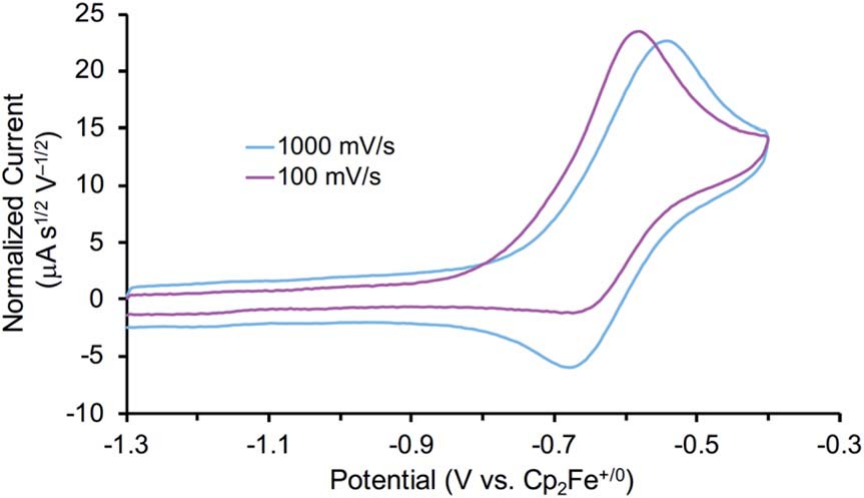
Cyclic voltammogram of the first oxidation of 3 (0.2 mM) in 0.2 M NBu_4_PF_6_ solution in THF under N_2_ using a glassy carbon working electrode, Pt wire auxiliary electrode, and Ag wire pseudoreference. Potentials referenced to Cp_2_Fe^+/0^ after the experiments.

In other tests, we explored whether a stepwise ET-PT pathway (electron transfer followed by proton transfer) is feasible. CV of 3 in THF shows an irreversible oxidation wave (*E*_pa_ = −0.61 V *vs.* Cp_2_Fe^+/0^) at a scan rate of 100 mV s^−1^ (Fig. 3). However, increasing the scan rate to 1 V s^−1^ results in a distinct anodic shift of the oxidation event and increased reversibility, indicating chemical follow-up steps at a time-scale of the CV experiment. This potential is similar to those for the oxidation of both the dichloride complex 1 and the Re(NH_3_)_2_ complex 4.^60^ Further analysis of the CV has not been fruitful because of the lack of reversibility and formation of unknown byproducts (see below). However, electrolysis of a solution of 3 in the presence of 2,6-lutidine (p*K*_a_ = 7.2 in THF^70^) at a potential of +0.6 V relative to the open circuit potential (OCP) resulted in steady passing of charge up to 2.2 equiv. e^−^ (Fig. S15†) and a change in color from brown to orange. Rhenium(v) nitride complex 5, in which the backbone is protonated,^66^ was isolated from the post-electrolysis mixture in 69% isolated yield (Fig. S17†). The combination of removing an electron at −0.61 V and a proton with lutidine is thermodynamically equivalent to an “effective BDFE” of 56 kcal mol^−1^,^69,71^ so this e^−^/H^+^ removal is thermodynamically similar to the HAA reactions above.

**Scheme 5:**
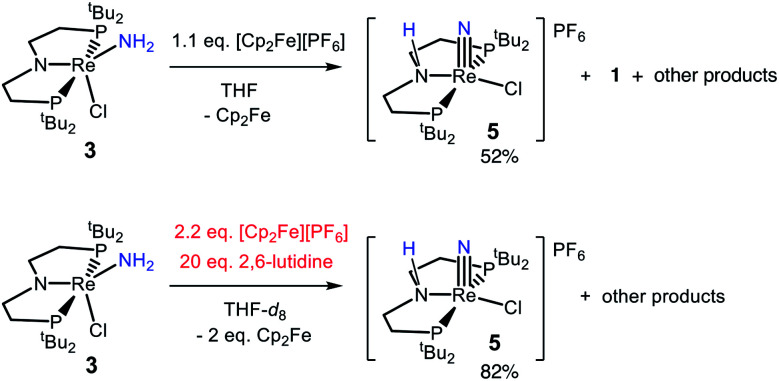
Reactions of 3 with chemical oxidants.

In an effort to identify oxidation products of 3, chemical oxidation was carried out with 1.1 equiv. of [Cp_2_Fe][PF_6_] in THF-*d*_8_ (Scheme 5). The major product identified from the resulting ^1^H and ^31^P{^1^H} NMR spectra was 5 in 50% yield (Fig. S9†).^66^ Furthermore, the Re^III^-dichloride complex 1 was obtained in 25% yield, as well as a brown precipitate that could not be identified. The formation of both Re^III^ and Re^V^ complexes from the 1e^−^ oxidation of 3 implies disproportionation; however, these products are not formed in a 1 : 1 ratio, implicating additional decomposition pathways. The product mixture that can be identified spectroscopically does not account for all of the Re, N, or H atoms present in the starting material. To test whether the missing H atoms could be released as H_2_ from weakened N–H bonds during the reaction, the reaction was repeated on a larger scale. Analysis of the THF-soluble products from the reaction showed formation of 1 and 5 in 17% and 52% yield, respectively, and no H_2_ was detected from analysis of the headspace (<1% yield).

We also tested whether oxidation of 3 to a nitride could be facilitated by providing an exogenous base for the deprotonation steps and by using 2 equiv. of oxidant (Scheme 5). Accordingly, 20 equiv. 2,6-lutidine and 2.2 equiv. [Cp_2_Fe][PF_6_] were added to a solution of 3 in THF-*d*_8_, forming 5 in 82% spectroscopic yield, and no observable 1 (Fig. S10†). An unidentified brown solid was formed as a byproduct in both reactions, suggesting decomposition. The formation of unknown byproducts deterred us from further mechanistic analysis.

### Calorimetric measurement and DFT calculations of N–H bond oxidation from (PNP)Re(NH_2_)Cl

Since the reaction of 3 with 2 equiv. ^*t*^Bu_3_PhO˙ to form 2 and 2 equiv. ^*t*^Bu_3_PhOH proceeds quantitatively, we chose this reaction for calorimetric determination of the reaction enthalpy.^72,73^ The titration of 3 with ^*t*^Bu_3_PhO˙ in THF using isothermal titration calorimetry (ITC) results in an exotherm of −50.9 kcal mol^−1^ until 2.0 equiv. of ^*t*^Bu_3_PhO˙ are added (Fig. S18†).^33,73^ Thus, on average, each abstraction of an H atom from 3 gives an enthalpy change of −25.4 kcal mol^−1^. Since the bond dissociation enthalpy of ^*t*^Bu_3_PhO–H in THF is 80.8 ± 1 kcal mol^−1^,‡‡The BDFE of ^*t*^Bu_3_PhO–H in THF has recently been reported as 74.4 kcal mol^−1^,^69^ so the bond dissociation enthalpy (BDE) can be estimated as 80.7 kcal mol^−1^. This takes *TS*° for H˙ in THF to be 6.3 ± 0.2 kcal mol^−1^, the mean of the *TS*°(H˙) values for moderately polar aprotic solvents. the average of the two BDE_N–H_ values of 3 in THF is 55.4 ± 1 kcal mol^−1^.

DFT calculations were used to obtain more insight into the thermodynamics of each PCET step from amide complex 3 to nitride complex 2. The B3LYP functional and def2-TZVP basis, together with standard solvent and dispersion corrections gave excellent agreement with the metrical parameters of the crystal structure of 3, and predicted the redox potential for 3^+/0^ (*E* = −0.65 V) close to the observed wave at −0.61 V in the experimental CV (see ESI†). Computation of the putative PCET intermediate confirmed that the *S* = 1/2 Re^IV^–imide (PNP)Re(NH)Cl (LRe=NH) is the most stable tautomer; an isomeric amidorhenium(iv) complex, (PNP*)Re(NH_2_)Cl (PNP* = N(CHCH_2_P^*t*^Bu_2_)(CH_2_CH_2_P^*t*^Bu_2_)), with an unsaturated PNP backbone proved higher in free energy by 12 kcal mol^−1^. The optimized structure of LRe=NH shows a strongly bent parent imido ligand with a computed Re–N–H angle of 133°. Bending reduces the antibonding π-interaction of the multiply bonding imido ligand with the metal centred SOMO after reduction. In consequence, the Re–imide bond is considerably elongated (1.80 Å) with respect to the parent nitride (DFT: 1.66 Å).

**Scheme 6:**
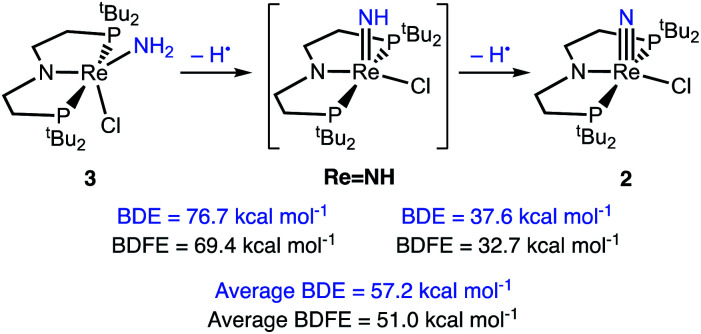
DFT (B3LYP/def2-TZVP) computed thermochemistry for the oxidation of 3 to 2*via* stepwise H-atom removal.

Computations of the *enthalpies* associated with each sequential H-atom transfer from 3 to ^*t*^Bu_3_PhO˙ to give ^*t*^Bu_3_PhOH in THF gave an average calculated BDE_N–H_ of 57 kcal mol^−1^ (Scheme 6).^38,40,58^ This is close to the calorimetrically determined average BDE of 55.4 ± 1 kcal mol^−1^. The *free energy* of conversion of 3 to 2*via* H-atom transfer to ^*t*^Bu_3_PhO˙ gave an average calculated BDFE_N–H_ in 3 of 51 kcal mol^−1^. Further insight comes from the hypothetical 1e^−^/1H^+^ steps. The BDFE_N–H_ for removing the first H from the amide ligand in 3 was calculated to be 69 kcal mol^−1^, which is substantially higher than the computed BDFE_N–H_ of the second N–H bond (in LRe=NH), 33 kcal mol^−1^. These computations show that the N–H bond in LRe=NH is particularly weak, which is consistent with both the facile, irreversible oxidation of 3 with HAA reagents and the inability to observe this putative parent imido complex (see ESI†).

## Discussion

### NH_3_ conversion to nitride with (PNP)Re

**Scheme 7:**
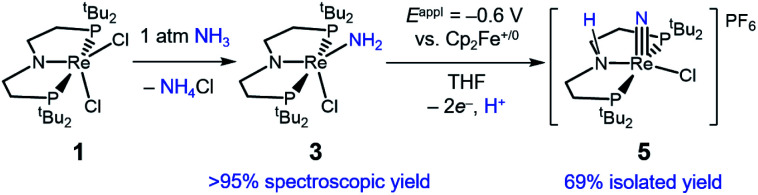
Full conversion of NH_3_ by 1 to a nitride in complex 5.

The conversion of NH_3_ to a nitride is quite facile in this system. Using excess NH_3_, complex 1 goes directly to the rhenium amide complex 3. The intermediate Re^III^-ammine complex, potentially (PNP)Re(NH_3_)Cl_2_, has been neither spectroscopically observed nor isolated, which we attribute to rapid dehydrohalogenation by free NH_3_. This can be avoided through preparation of the cationic Re^III^(NH_3_)_2_ complex 4, which can be deprotonated to form isolable complex 3. Further oxidation of 3 to Re–nitride complexes is also facile, forming 2 using either hydrogen atom abstracting (HAA) reagents or forming 5*via* 2e^−^ chemical oxidation in the presence of a weak base. Thus, the complete conversion of NH_3_ to a nitride in this system is achievable in good yield using 2e^−^ electrochemical oxidation in the presence of base (Scheme 7). From a functional standpoint, such facile formation of a nitride complex from NH_3_ is attractive considering the mild oxidation potential used (−0.6 V) and the possibility for excess NH_3_ to serve as an exogenous base.^81^ However, turnover to achieve catalytic NH_3_ oxidation to N_2_ would require a nitride coupling step.^35,82,83^ Although nitride coupling reactions between other late transition-metal nitrides bearing similar supporting pincer ligands have been reported,^46,84–86^ nitride coupling is not observed in this system due to the strong Re–nitride bond in complex 2.^66^ We attribute this to a thermodynamic difficulty because the reverse reaction, reductive N_2_ cleavage to form 2, is very exergonic.

The calorimetric titrations give an average bond enthalpy for the two N–H bonds in 3 of 55.4 ± 1 kcal mol^−1^ in THF. This is a rare example of an experimentally-derived bond energy in the context of NH_3_ oxidation.^74^ In contrast, almost all other literature values (Table 2; see also ref. 45 and 75) are estimated through bracketing experiments or computational models.^32^ The computationally derived average BDE_N–H_ of 57 kcal mol^−1^ agrees with the experimental value, and the computations indicate that the analogous average BDFE (free energy) is 51 kcal mol^−1^. This value represents a substantial weakening from the BDFE_N–H_ of free NH_3_ (100 kcal mol^−1^).^8^

**Table 2:** Comparison of the measured average N–H bond dissociation enthalpy in 3 to computed N–H bond dissociation free energies (BDFE) of NH_*x*_ ligands in other relevant systems

Complex	Solvent	BDFE_N–H_ (kcal mol^−1^)			Reference
		NH_3_	NH_2_	NH	
(PNP)Re(NH*_x_*)Cl (3, computed)	THF	—	69	33	This work
(PNP)Re(NH*_x_*)Cl (3, experimental)	THF		57(ave)^a^		This work
*cis*-(PONOP)Re(NH*_x_*)Cl_2_	THF	—	78	43	58
(PNP)Ir(NH*_x_*)	Gas phase	—	95^a^	71^a^	46
*trans*-[(Ph-tpy)(PPh_2_Me)_2_Mo(NH*_x_*)]^+^	THF	46	64	—	74 and 75
*cis*-[(Cp)(P^Ph^_2_N^tBu^_2_)Mo(NH*_x_*)(CO)]^+^	Et_2_O	84	61	—	38
[(PY5)Mo(NH*_x_*)]^2+^	MeCN	68	65	64	40
[(Cp*)(P^tBu^_2_N^Ph^_2_)Ru(NH*_x_*)]^+^	THF	83	89	72	43
[(tpy)(^NMe2^bpy)Ru(NH*_x_*)]^2+^	THF	79	86	—	56
(TMP)Ru(NH*_x_*)_2_	C_6_H_6_	82	93	75	45
[(tpy)(^NMe2^bpy)Fe(NH*_x_*)]^2+^	THF	82	90	—	56
[(^Ph^NCH_2_CH_2_)_3_N]Mo(NH*_x_*)	—	52	64	42	76
[(BP_3_)Fe(NH*_x_*)]^+^	Et_2_O	—	80	65	77
(F)(H_2_PCH_2_CH_2_PH_2_)_2_Mo(NH*_x_*)	Benzene	41	92	37	78
(salen)Mn(NH*_x_*)	Gas phase	85	84	60	79
(*η*^5^-C_5_Me_4_SiMe_3_)_2_Ti(NH*_x_*)	Gas phase	42	79	—	80

a Bond dissociation enthalpies (BDE).

The oxidation of ammine complexes to form nitrides using HAA reagents is precedented in other systems using ^*t*^Bu_3_PhO˙ as the oxidant (forming ^*t*^Bu_3_PhOH).^37–40,43,46^ H-atom abstraction from the amide in 3 can be achieved with HAA reagents to form X–H bonds that are up to 15 kcal mol^−1^ weaker than the O–H bond in ^*t*^Bu_3_PhOH, though the phenazine reaction requires heating. The first HAA from 3 using organic reagents can be thermodynamically unfavorable by up to 10 kcal mol^−1^ because nitride formation is driven by the much more favorable second N–H oxidation.^37,38,45^

Though we have no experimental evidence for the intermediate imido complex LRe=NH, we considered its properties obtained from a DFT model. These computations indicate that the N–H bond in LRe=NH is especially weak, with a BDFE of 33 kcal mol^−1^. This bond is 36 kcal mol^−1^ weaker than the calculated first BDFE_N–H_ of amido complex 3. Additionally, the N–H bond in LRe=NH is calculated to be 10 kcal mol^−1^ weaker than the 43 kcal mol^−1^ value computed for the closely related (PONOP)Re^IV^=NH (PONOP = 2,6-bis-(diisopropylphosphinito)pyridine).^58^

As this last example shows, the thermochemistry of N–H bond forming and breaking is of particular interest for understanding how to achieve efficient N_2_ to NH_3_ interconversion. Table 2 compares our experimental values to literature values, typically derived from computational modelling. Complex 3 and its analogue (PONOP)Re(NH_2_)(Cl)_2_, both pincer-supported Re^III^–NH_2_ complexes, have low BDFE_N–H_ values. Interestingly, the Re^IV^=NH compounds exhibit weaker imide N–H bonds than those of other metals. Some of the weakest N–H bonds were calculated for Mo–NH intermediates in the Chatt^78^ (37 kcal mol^−1^) and Schrock^76^ (42 kcal mol^−1^) systems, which can undergo complete N_2_ reduction to ammonia.^79^ Consistent with earlier systems (Table 2), amide intermediates consistently exhibit stronger N–H bonds than their corresponding imide intermediates. However, the difference between these two bond energies is particularly large in the Re systems, especially 3 where ΔBDFE_N–H_ = 36 kcal mol^−1^. These values can be qualitatively rationalized by the very strong Re- and Mo-nitride bonds that arise when a *d*^2^ configuration is reached, and the less favorable M–N π bonding at higher d-electron counts.

### Relevance to the PCET nitride reduction step of NRR

Nitride complex 2 is readily formed *via* electrochemical N_2_ cleavage,^60^ so the conversion of NH_3_ to the nitride ligand in 2 represents part of the reverse pathway from N_2_ to NH_3_ (Scheme 1). This would involve N_2_ cleavage to form 2 followed by 3e^−^/3H^+^ PCET reduction of the nitride. In a recent review, Chirik and coworkers highlighted the lack of data in the literature on the bond strengths of N–H bonds in NH_2_ and NH complexes in systems that perform N_2_ reduction,^76^ and the studies here are an important step toward understanding these species quantitatively. The thermochemical data from this study identify specific challenges associated with steps during the conversion of N_2_ to NH_3_.

One clear challenge in the PCET reduction of 2 is formation of the first N–H bond, which would give a very weak bond in LRe=NH with a BDFE_N–H_ of only 33 kcal mol^−1^.^76,78,79^ One approach that has been used to overcome this difficulty in literature systems is the use of potent acid/reductant pairs to form the weak N–H bonds,^21,29,30,58,79,87^ though this is complicated in the current system by the ease of protonation of the pincer amide group.^66^ In general, the instability of the imide species is identified as a key hindrance, because the imide intermediate must be accessed, even transiently, on the way to subsequent reduction that gives the amide species. The low driving force for PCET-assisted reduction of nitride 2 can be attributed to an overstabilization that results from strong Re≡N triple bonding.

Besides these thermochemical considerations, an encouraging result is that the amide complex 3 can be readily oxidized even though the first step (formation of Re=NH) is uphill. The conversion of 3 to 2 by TEMPO, for instance, occurs within minutes even though the first step is thermodynamically uphill by 3 kcal mol^−1^. The ability of the first H˙ abstraction to proceed indicates that the barriers for the H-atom transfer reactions are low. Therefore, even less stable imide intermediates may be sufficient to allow rapid catalysis in the future.

## Conclusions

Rhenium-amide and -ammine complexes have been isolated, and they are readily oxidized to the corresponding metal–nitride complex. The conversion of NH_3_ to a nitride by Re(PNP)Cl_2_ (1) to form Re(PNP)(N)Cl (2) represents the first example of an NH_3_-to-nitride transformation at Re. Since 2 can also be generated *via* electrochemical N_2_ cleavage, this system is relevant both to N_2_ reduction and ammonia oxidation. Facile, initial deprotonation of NH_3_ occurs upon coordination to 1 to form the amide complex Re(PNP)(NH_2_)Cl (3). The subsequent conversion to Re≡N can be accomplished with weak hydrogen atom acceptors that provide low driving force for the H˙ transfer, indicating that the reactions are kinetically facile.

Calorimetric measurements of the conversion of 3 to 2 with ^*t*^Bu_3_PhO˙ show that the average BDE_N–H_ of 3 in THF is 55.4 ± 1 kcal mol^−1^. This is a rare experimental thermochemical measurement of N–H bond strengths relevant to N_2_/NH_3_ interconversions.^76^ Computations show that this average is derived from two disparate N–H bond strengths, as the second N–H BDE in 3 (77 kcal mol^−1^) is much stronger than the N–H BDE in the putative imide intermediate LRe^IV^=NH (38 kcal mol^−1^). The ability to form the weak N–H bond in this imide, even transiently, is identified as a crucial bottleneck for N_2_ to NH_3_ conversion in this system. The combination of bracketing,^46^ calorimetry and DFT is a powerful strategy that will continue to elucidate the steps of N_2_ reduction at metal complexes.

## Data availability

Crystallographic data have been deposited at the CCDC with deposition numbers 2100974-2100975 and can be obtained from https://www.ccdc.cam.ac.uk/structures/. Other data supporting this article have been uploaded as part of the ESI.†

## Author contributions

Conceptualization: G. P. C., J. M. M., P. L. H.; investigation and analysis: G. P. C., D. D., J. E. W., B. Q. M., J. B. C.; writing, interpretation: G. P. C., D. D., J. E. W., S. S., J. M. M., P. L. H.

## Conflicts of interest

There are no conflicts to declare.

## Supplementary Material

SC-013-D1SC04503B-s001

SC-013-D1SC04503B-s002

## References

[cit1] Smil V. , Enriching the Earth: Fritz Haber, Carl Bosch, and the Transformation of World Food Production, MIT Press, Cambridge, MA, 2004

[cit2] Erisman J. W., Sutton M. A., Galloway J., Klimont Z., Winiwarter W. (2008). Nat. Geosci..

[cit3] Renner J. N., Greenlee L. F., Ayres K. E., Herring A. M. (2015). Electrochem. Soc. Interface.

[cit4] Service R. F. (2018). Science.

[cit5] Martin A. J., Shinagawa T., Perez-Ramirez J. (2019). Chem.

[cit6] Adli N. M., Zhang H., Mukherjee S., Wu G. (2018). J. Electrochem. Soc..

[cit7] Elishav O., Mosevitzky Lis B., Miller E. M., Arent D. J., Valera-Medina A., Grinberg Dana A., Shter G. E., Grader G. S. (2020). Chem. Rev..

[cit8] Warren J. J., Tronic T. A., Mayer J. M. (2010). Chem. Rev..

[cit9] Weinberg D. R., Gagliardi C. J., Hull J. F., Murphy C. F., Kent C. A., Westlake B. C., Paul A., Ess D. H., McCafferty D. G., Meyer T. J. (2012). Chem. Rev..

[cit10] Hochman G., Goldman A. S., Felder F. A., Mayer J. M., Miller A. J. M., Holland P. L., Goldman L. A., Manocha P., Song Z., Aleti S. (2020). ACS Sustainable Chem. Eng..

[cit11] Rebreyend C., de Bruin B. (2015). Angew. Chem., Int. Ed..

[cit12] van der Ham C. J. M., Koper M. T. M., Hetterscheid D. G. H. (2014). Chem. Soc. Rev..

[cit13] Rosca V., Duca M., de Groot M. T., Koper M. T. M. (2009). Chem. Rev..

[cit14] Kyriakou V., Garagounis I., Vasileiou E., Vourros A., Stoukides M. (2017). Catal. Today.

[cit15] Liu H., Wei L., Liu F., Pei Z., Shi J., Wang Z.-J., He D., Chen Y. (2019). ACS Catal..

[cit16] Wan Y., Xu J., Lv R. (2019). Mater. Today.

[cit17] Yandulov D. V., Schrock R. R. (2003). Science.

[cit18] Kuriyama S., Arashiba K., Nakajima K., Tanaka H., Kamaru N., Yoshizawa K., Nishibayashi Y. (2014). J. Am. Chem. Soc..

[cit19] Arashiba K., Kinoshita E., Kuriyama S., Eizawa A., Nakajima K., Tanaka H., Yoshizawa K., Nishibayashi Y. (2015). J. Am. Chem. Soc..

[cit20] Arashiba K., Eizawa A., Tanaka H., Nakajima K., Yoshizawa K., Nishibayashi Y. (2017). Bull. Chem. Soc. Jpn..

[cit21] Ashida Y., Arashiba K., Nakajima K., Nishibayashi Y. (2019). Nature.

[cit22] Arashiba K., Miyake Y., Nishibayashi Y. (2011). Nat. Chem..

[cit23] Wickramasinghe L. A., Ogawa T., Schrock R. R., Muller P. (2017). J. Am. Chem. Soc..

[cit24] Itabashi T., Mori I., Arashiba K., Eizawa A., Nakajima K., Nishibayashi Y. (2019). Dalton Trans..

[cit25] Eizawa A., Arashiba K., Egi A., Tanaka H., Nakajima K., Yoshizawa K., Nishibayashi Y. (2019). Chem.–Asian J..

[cit26] Anderson J. S., Rittle J., Peters J. C. (2013). Nature.

[cit27] Creutz S. E., Peters J. C. (2013). J. Am. Chem. Soc..

[cit28] Del Castillo T. J., Thompson N. B., Peters J. C. (2016). J. Am. Chem. Soc..

[cit29] Chalkley M. J., Del Castillo T. J., Matson B. D., Roddy J. P., Peters J. C. (2017). ACS Cent. Sci..

[cit30] Chalkley M. J., Del Castillo T. J., Matson B. D., Peters J. C. (2018). J. Am. Chem. Soc..

[cit31] Chalkley M. J., Drover M. W., Peters J. C. (2020). Chem. Rev..

[cit32] Dunn P. L., Cook B. J., Johnson S. I., Appel A. M., Bullock R. M. (2020). J. Am. Chem. Soc..

[cit33] Dehnicke K., Strähle J. (1992). Angew. Chem., Int. Ed. Engl..

[cit34] Du Bois J., Hong J., Carreira E. M., Day M. W. (1996). J. Am. Chem. Soc..

[cit35] Clarke R. M., Storr T. (2016). J. Am. Chem. Soc..

[cit36] Keener M., Peterson M., Hernandez Sanchez R., Oswald V. F., Wu G., Menard G. (2017). Chem. –Eur. J..

[cit37] Cook B. J., Johnson S. I., Chambers G. M., Kaminsky W., Bullock R. M. (2019). Chem. Commun..

[cit38] Bhattacharya P., Heiden Z. M., Wiedner E. S., Raugei S., Piro N. A., Kassel W. S., Bullock R. M., Mock M. T. (2017). J. Am. Chem. Soc..

[cit39] Margulieux G. W., Bezdek M. J., Turner Z. R., Chirik P. J. (2017). J. Am. Chem. Soc..

[cit40] Johnson S. I., Heins S. P., Klug C. M., Wiedner E. S., Bullock R. M., Raugei S. (2019). Chem. Commun..

[cit41] Pipes D. W., Bakir M., Vitols S. E., Hodgson D. J., Meyer T. J. (1990). J. Am. Chem. Soc..

[cit42] Coia G. M., Demadis K. D., Meyer T. J. (2000). Inorg. Chem..

[cit43] Bhattacharya P., Heiden Z. M., Chambers G. M., Johnson S. I., Bullock R. M., Mock M. T. (2019). Angew. Chem., Int. Ed..

[cit44] Nakajima K., Toda H., Sakata K., Nishibayashi Y. (2019). Nat. Chem..

[cit45] Dunn P. L., Johnson S. I., Kaminsky W., Bullock R. M. (2020). J. Am. Chem. Soc..

[cit46] Scheibel M. G., Abbenseth J., Kinauer M., Heinemann F. W., Wuertele C., de Bruin B., Schneider S. (2015). Inorg. Chem..

[cit47] Thusius D. D., Taube H. (1967). J. Phys. Chem..

[cit48] Taube H., White J. D. (1970). J. Phys. Chem..

[cit49] Buhr J. D., Taube H. (1979). Inorg. Chem..

[cit50] Collman J. P., Hutchison J. E., Ennis M. S., Lopez M. A., Guilard R. (1992). J. Am. Chem. Soc..

[cit51] Ishitani O., White P. S., Meyer T. J. (1996). Inorg. Chem..

[cit52] Ishitani O., Ando E., Meyer T. J. (2003). Inorg. Chem..

[cit53] Zott M. D., Garrido-Barros P., Peters J. C. (2019). ACS Catal..

[cit54] Habibzadeh F., Miller S. L., Hamann T. W., Smith III M. R. (2019). Proc. Natl. Acad. Sci. U. S. A..

[cit55] Zott M. D., Peters J. C. (2021). J. Am. Chem. Soc..

[cit56] Najafian A., Cundari T. R. (2019). J. Phys. Chem. A.

[cit57] Forrest S. J. K., Schluschaß B., Yuzik-Klimova E. Y., Schneider S. (2021). Chem. Rev..

[cit58] Bruch Q. J., Connor G. P., Chen C.-H., Holland P. L., Mayer J. M., Hasanayn F., Miller A. J. M. (2019). J. Am. Chem. Soc..

[cit59] Klopsch I., Finger M., Wuertele C., Milde B., Werz D. B., Schneider S. (2014). J. Am. Chem. Soc..

[cit60] Lindley B. M., van Alten R. S., Finger M., Schendzielorz F., Würtele C., Miller A. J. M., Siewert I., Schneider S. (2018). J. Am. Chem. Soc..

[cit61] van Alten R. S., Wätjen F., Demeshko S., Miller A. J. M., Würtele C., Siewert I., Schneider S. (2020). Eur. J. Inorg. Chem..

[cit62] Klopsch I., Kinauer M., Finger M., Würtele C., Schneider S. (2016). Angew. Chem., Int. Ed..

[cit63] Klopsch I., Schendzielorz F., Volkmann C., Würtele C., Schneider S. (2018). Z. Anorg. Allg. Chem..

[cit64] Earnshaw A., Figgis B. N., Lewis J., Peacock R. D. (1961). J. Chem. Soc..

[cit65] Chatt J., Leigh G. J., Mingos D. M. P. (1969). J. Chem. Soc. A.

[cit66] Connor G. P., Mercado B. Q., Lant H. M. C., Mayer J. M., Holland P. L. (2019). Inorg. Chem..

[cit67] Hillhouse G. L., Bercaw J. E. (1984). J. Am. Chem. Soc..

[cit68] Lindley B. M., Bruch Q. J., White P. S., Hasanayn F., Miller A. J. M. (2017). J. Am. Chem. Soc..

[cit69] Wise C. F., Agarwal R. G., Mayer J. M. (2020). J. Am. Chem. Soc..

[cit70] Tshepelevitsh S., Kütt A., Lõkov M., Kaljurand I., Saame J., Heering A., Plieger P. G., Vianello R., Leito I. (2019). Eur. J. Org. Chem..

[cit71] Waidmann C. R., Miller A. J. M., Ng C.-W. A., Scheuermann M. L., Porter T. R., Tronic T. A., Mayer J. M. (2012). Energy Env. Sci..

[cit72] Delony D., Kinauer M., Diefenbach M., Demeshko S., Würtele C., Holthausen M. C., Schneider S. (2019). Angew. Chem., Int. Ed..

[cit73] Abbenseth J., Delony D., Neben M. C., Würtele C., de Bruin B., Schneider S. (2019). Angew. Chem., Int. Ed..

[cit74] Bezdek M. J., Guo S., Chirik P. J. (2016). Science.

[cit75] Bezdek M. J., Chirik P. J. (2018). Angew. Chem., Int. Ed..

[cit76] Bezdek M. J. , Pappas I. and Chirik P. J., in Nitrogen Fixation, ed. Y. Nishibayashi, Springer International Publishing, Cham, 2017, pp. 1–21, 10.1007/3418_2016_8

[cit77] Matson B. D., Peters J. C. (2018). ACS Catal..

[cit78] Stephan G. C., Sivasankar C., Studt F., Tuczek F. (2008). Chem. –Eur. J..

[cit79] Wang D., Loose F., Chirik P. J., Knowles R. R. (2019). J. Am. Chem. Soc..

[cit80] Pappas I., Chirik P. J. (2016). J. Am. Chem. Soc..

[cit81] Adli N. M., Zhang H., Mukherjee S., Wu G. (2018). J. Electrochem. Soc..

[cit82] Keener M., Peterson M., Hernandez Sanchez R., Oswald V. F., Wu G., Menard G. (2017). Chem. –Eur. J..

[cit83] Man W.-L., Tang T.-M., Wong T.-W., Lau T.-C., Peng S.-M., Wong W.-T. (2004). J. Am. Chem. Soc..

[cit84] Scheibel M. G., Wu Y., Stückl A. C., Krause L., Carl E., Stalke D., de Bruin B., Schneider S. (2013). J. Am. Chem. Soc..

[cit85] Abbenseth J., Finger M., Wuertele C., Kasanmascheff M., Schneider S. (2016). Inorg. Chem. Front..

[cit86] Scheibel M. G., Askevold B., Heinemann F. W., Reijerse E. J., de Bruin B., Schneider S. (2012). Nature Chem..

[cit87] Chalkley M. J., Oyala P. H., Peters J. C. (2019). J. Am. Chem. Soc..

