# Supplementary material for: Facile conversion of ammonia to a nitride in a rhenium system that cleaves dinitrogen
Source: Chem Sci. 2022 Mar 4;13(14):4010–8. doi: 10.1039/d1sc04503b (PMC8985503; doi:10.1039/d1sc04503b)
Supplement: SC-013-D1SC04503B-s001 [file SC-013-D1SC04503B-s001.pdf]

Electronic Supplementary Material (ESI) for Chemical Science.
This journal is © The Royal Society of Chemistry 2022

Supporting Information for

Facile Conversion of Ammonia to a Nitride in a Rhenium System that Cleaves Dinitrogen

Gannon P. Connor, Daniel Delony, Jeremy E. Weber, Brandon Q. Mercado, Julia B. Curley, Sven Schneider, James M. Mayer, and Patrick L. Holland**

Department of Chemistry, Yale University, New Haven, Connecticut, USA, and

Department of Inorganic Chemistry, Georg-August-Universität Göttingen, Göttingen, Germany

Table of Contents

Experimental	S-2
Preparation of synthetic compounds	S-3
Analytical methods	S-6
NMR spectra	S-8
IR spectra	S-18
UV-vis spectra	S-20
Electrochemistry	S-22
Calorimetry	S-25
Computations	S-28
Crystallography	S-37
References	S-41

Experimental

All manipulations were performed under an inert atmosphere of N_2 gas in a M. Braun glovebox or on a Schlenk line unless otherwise specified. Tetrahydrofuran (THF) was distilled under argon from potassium benzophenone ketyl and stored over molecular sieves prior to use. Unless otherwise noted, all other solvents were dried via passage through Q5 columns from Glass Contour Co. and stored over molecular sieves prior to use. Deuterated solvents were degassed and dried over calcium hydride before storing over molecular sieves prior to use, and THF-d_8 was dried additionally with potassium benzophenone ketyl. Deionized water was degassed prior to use. Anhydrous ammonia (Airgas, >99.9%), potassium hexamethyldisilazide (Sigma-Aldrich, 95%, recrystallized), decamethyl cobaltocene (Sigma-Aldrich, 95%, recrystallized), ferrocenium hexafluorophosphate (Santa Cruz Biotechnology, 97%), 2,6-lutidine (Sigma-Aldrich, 98%, distilled and dried over sieves), 2,4,6-tri-*tert*-butylphenol (Sigma-Aldrich, 97%), TEMPO$^•$ (Sigma-Aldrich, 98%), phenazine (Sigma-Aldrich, 98%), azobenzene (Sigma-Aldrich, 98%), 1,8-diazabicyclo[5.4.0]undec-7-ene (Sigma-Aldrich, 98%, distilled and dried over sieves), phosphazene base P_1-tBu-tris(tetramethylene) (Sigma-Aldrich, dried under vacuum), phosphazene base P_4-tBu (Sigma-Aldrich, dried under vacuum), trimethylphosphine (Sigma-Aldrich, 1 M in THF), and tetrabutylammonium hexafluorophosphate (Sigma-Aldrich, 99%, recrystallized three times) were purchased and used without further purification unless otherwise noted. The synthesis and purification for the following reagents used literature procedures: $SPMe_3$,[1] $^tBu_3PhO^•$,[2] 1,8-dichloro-9,10-anthracenediol,[3] (PNP)$ReCl_2$ (**1**).[4]

NMR spectra were acquired on an Agilent 400 MHz or 500 MHz spectrometer. ^1H spectra were referenced to residual ^1H signals from the deuterated solvent with which the sample was prepared,[5] and $^{13}C\{^1H\}$, $^{31}P\{^1H\}$, and ^{15}N spectra were absolute referenced to the corresponding

^1H spectra. IR spectra were obtained using a Bruker Alpha spectrometer containing a diamond ATR unit with 2 cm^{-1} resolution.

Electrochemical measurements were collected using a CH Instruments 600D potentiostat in an Ar glovebox. Cyclic voltammetry (CV) experiments were conducted in 0.2 M tetrabutylammonium hexafluorophosphate (TBA-PF_6) solutions in dry solvent using a glassy carbon disc working electrode (3.0 mm diameter), a platinum wire auxiliary electrode, and a silver wire psuedoreference. Wire electrodes were sanded and rinsed with dry solvent prior to use, and glassy carbon disc electrodes were polished using 0.05 µm alumina powder and rinsed with dry solvent prior to use. Measurements conducted in THF were compensated for internal resistance prior to collecting data. CV experiments were referenced to ferrocene (Cp_2Fe) as an internal standard after completion.

Elemental analyses were obtained from the CENTC Elemental Analysis Facility at the University of Rochester. Microanalysis samples were weighed on a PerkinElmer Model AD-6 Autobalance, analyzed on a PerkinElmer 2400 Series II Analyzer, and handled in a VAC Atmospheres glovebox under argon.

Preparation of (PNP)Re(NH_2)(Cl) (3): A sample of **1** (283.2 mg, 0.459 mmol) was dissolved in benzene (20 mL) under Ar in a resealable Schlenk tube. The solution was degassed and warmed to ambient temperature, and the atmosphere was replaced with NH_3 gas (1 atm) while stirring. There was an immediate color change of the solution from purple to brown. The mixture was stirred for 1 min, then frozen using a liquid N_2 bath. The flask was placed in an ice water bath, and the benzene was lyophilized at 0 °C to give a light brown powder. The powder was extracted with pentane (3 x 10 mL), and the mixture was filtered through Celite to remove a pale blue solid (109.3 mg). Drying the filtrate under vacuum gave **3** as a brown microcrystalline solid (167.6 mg,

0.280 mmol, 61% yield). Crystals of **3** suitable for XRD grew from a concentrated pentane solution at –40 °C. ^{1}H NMR (400 MHz, C_6D_6): δ 12.70 (br s, 2H, N*H*$_2$), 3.59 (m, 2H, N(C*H*$_2$CH$_2$)$_2$), 3.34 (m, 2H, N(C*H*$_2$CH$_2$)$_2$), 1.89 (m, 2H, N(CH$_2$C*H*$_2$)$_2$), 1.60 (m, 2H, N(CH$_2$C*H*$_2$)$_2$), 1.36 (vt, 18H, P(*^{t}Bu*)(^{t}Bu')), 1.20 (vt, 18H, P(^{t}Bu)(*^{t}Bu'*)) ppm. $^{31}P\{^{1}H\}$ NMR (162 MHz, C_6D_6) δ 40.08 (s) ppm. ^{15}N NMR (51 MHz, C_6D_6): δ –260 (s, *N*H$_2$) ppm. UV-vis (THF solution): 308 nm (ε = 5600 M^{-1} cm^{-1}), 420 nm (ε = 1100 M^{-1} cm^{-1}), 562 nm (ε = 340 M^{-1} cm^{-1}). FTIR (solid, N–H): 3397, 3338 cm^{-1}. Anal. Calcd.(found) for $C_{20}H_{46}ClN_2P_2Re$ (%): C, 40.16(40.45); H, 7.75(7.74); N, 4.68(4.52).

Preparation of [*trans*-(PNP)Re(NH_3)$_2$(Cl)][BAr$^F_4$] (4): A sample of **1** (50.0 mg, 0.081 mmol, 1.0 equiv) and 71.7 mg NaBAr$^F_4$ (71.7 mg, 0.081 mmol, 1.0 equiv) was dissolved in THF (10 mL) in a resealable Schlenk tube under N_2. The N_2 atmosphere was removed with 3 freeze-pump-thaw cycles. The flask was then filled with anhydrous NH_3 (1 atm) while stirring at ambient temperature. There was an immediate color change of the solution from purple to light brown (almost colorless). The mixture was stirred for 1 min. The solvent and excess NH_3 were removed under vacuum to give a green residue. The residue was washed with pentane (3 x 5 mL), and the residue was extracted with THF (2 x 5 mL). Crystallization from a layered THF:pentane solution (12:4 mL) at –40 °C gave a pale green crystalline solid. The solid was collected and washed with benzene (3 x 3 mL), then dried under vacuum to give 54.5 mg of **4** as pale green crystals (0.368 mmol, 45.5% yield). ^{1}H NMR (400 MHz, THF-*d*$_8$): δ 7.77 (s, 8H, *o*-BAr$^F_4$), 7.56 (s, 4H, *p*-BAr$^F_4$), 5.47 (br s, 6H, N*H*$_3$), 4.49 (m, 4H, N(C*H*$_2$CH$_2$)$_2$), 2.04 (m, 4H, N(CH$_2$C*H*$_2$)$_2$), 1.29 (vt, 36H, P(*^{t}Bu*)$_2$) ppm. $^{31}P\{^{1}H\}$ NMR (162 MHz, THF-*d*$_8$): δ –9.98 (s) ppm. $^{19}F\{^{1}H\}$ NMR (376 MHz, THF-*d*$_8$): δ –63.5 (s, BAr$^F_4$) ppm. UV-Vis (THF solution): 328 nm (shoulder, ε = 3700 M^{-1} cm^{-1}). FTIR (solid, N–H): 3392, 3353, 3245, 3174 cm^{-1}. Anal. Calcd.(found) for $C_{52}H_{62}BF_{24}ClN_2P_2Re$ (%): C, 42.22(42.54); H, 4.22(4.16); N, 2.84(2.59).

H_2 quantification from 1e^- oxidation of (PNP)Re(NH_2)(Cl) (3): Under N_2, 54 mg $FcPF_6$ (0.16 mmol, 1.1 equiv) was slurried in 2 mL THF in a 7 mL scintillation vial. The vial was sealed with a septum cap, and a solution of 89 mg (PNP)Re(NH_2)(Cl) (**2**, 0.15 mmol, 1.0 equiv) in 2 mL THF was injected into the vial via syringe. An immediate color change from deep blue to orange occurred, as well as formation of a brown precipitate. The reaction was stirred for 10 min at ambient temperature, then chilled for 5 min in a dry ice/isopropanol bath to remove THF vapor from the headspace. 50 μL CH_4 gas was added via airtight syringe into the headspace of the vial as an internal standard, and 50 μL of the headspace of the vial was removed via airtight syringe for H_2 quantification on the GC. No H_2 peak was observed in the resulting chromatograph, indicating less than 1% yield of H_2. The reaction was repeated, with identical results.

Removal of the volatile materials under vacuum gave a sticky, dark red-orange residue. The residue was extracted with 2 x 5 mL THF and filtered through Celite. Removal of THF from the orange-brown filtrate under vacuum yielded a dirty orange solid. The solid was redissolved in ~10 mL THF, from which a ~2 mL aliquot was taken. The aliquot was dried under vacuum, dissolved in 0.7 mL THF-d_8, and analyzed using NMR spectroscopy vs. a 1,3,5-trismethoxybenzene (TMB) internal standard to quantify the Re products. Analysis of the ^{1}H NMR spectra showed 52% yield of nitride **5** and 17% yield of dichloride **1** for the first run, and 47% yield of nitride **5** and 20% yield of dichloride **1** for the second run.

H_2 quantification from the 1e^- reduction of [(PNP)Re(NH_3)$_2$(Cl)][BAr$^F_4$] (4): Under N_2, 174 mg of [(PNP)Re(NH_3)$_2$(Cl)][BAr$^F_4$] (0.12 mmol) was dissolved in 2 mL THF in a 7 mL scintillation vial. The vial was sealed with a septum cap, and a solution of 46 mg Cp^*_2Co (0.14 mmol, 1.1 equiv) dissolved in 2 mL THF was injected into the vial via syringe. An immediate color change from light tan to dark brown-gold occurred, as well as formation of a yellow

precipitate. The reaction was stirred for 10 min at ambient temperature, then chilled for 5 min in a dry ice/isopropanol bath to remove THF vapor from the headspace. 50 μL CH_4 gas was added via airtight syringe into the headspace of the vial as an internal standard, and 50 μL of the headspace of the vial was removed via airtight syringe for H_2 quantification on the GC. Gas chromatography used a ThermoFisher Trace 1300 GC apparatus with a thermal conductivity detector and a Supelco fused silica capillary column (5 Å molecular sieves, 30 m x 0.53 mm), and N_2 as a carrier gas. No H_2 peak was observed in the resulting chromatograph, indicating less than 1 ppm (1% yield) of H_2. The reaction was repeated, with identical results.

Removal of the volatile materials under vacuum gave a sticky, dark brown residue. The residue was extracted with 2 x 5 mL THF and filtered through Celite. Removal of THF from the brown filtrate under vacuum yielded a sticky brown solid. The solid was redissolved in ~10 mL THF, from which a ~2 mL aliquot was taken. The aliquot was dried under vacuum, dissolved in 0.7 mL THF-d_8, and analyzed using NMR spectroscopy vs. a 1,3,5-trimethoxybenzene (TMB) internal standard to quantify the Re products. Analysis of the ^{1}H NMR spectra showed formation of **3** in 73% for the first run and 61% for the second run. Decamethylcobaltocenium was also observed, though the peak was broadened and shifted from rapid self-exchange with Cp^*_2Co (Figure S5B).

Electrolysis of (PNP)Re(NH_2)(Cl) (3): In an Ar glovebox, an H-cell was prepared with the analyte chamber containing a solution of 20 mg of **3** (3.3×10^{-5} mol, 1.7 mM) and 77 μL of 2,6-lutidine (6.6×10^{-4} mol, 34 mM) in 20 mL of 0.2 M tetrabutylammonium hexafluorophosphate in THF, and the counter chamber containing a solution of 24.4 mg of [Cp_2Fe][PF_6] (7.4×10^{-5} mol, 3.7 mM, 2.2 equiv) in 20 mL of 0.2 M tetrabutylammonium hexafluorophosphate in THF. The analyte chamber was fitted with a carbon paper electrode and Ag wire pseudoreference, and the

counter chamber with a Pt mesh counter electrode. Under these conditions, the $E_{1/2}$ for the oxidation wave was +0.63 V vs. the open-circuit potential, and the potential of $E_{1/2}$ was chosen for the electrolysis. The potential of the analyte cell was held at $E_{1/2}$ for the oxidation, with both chambers gently stirring. Over the course of the electrolysis, the solution in the analyte chamber changed color from brown to orange, and the solution in the counter chamber changed color from blue to yellow. The electrolysis was stopped after 7.09 C of charge had passed (2.2 e^{-} per Re). The time vs. charge plot showed relatively constant passing of charge over the course of the electrolysis (Figure S15).

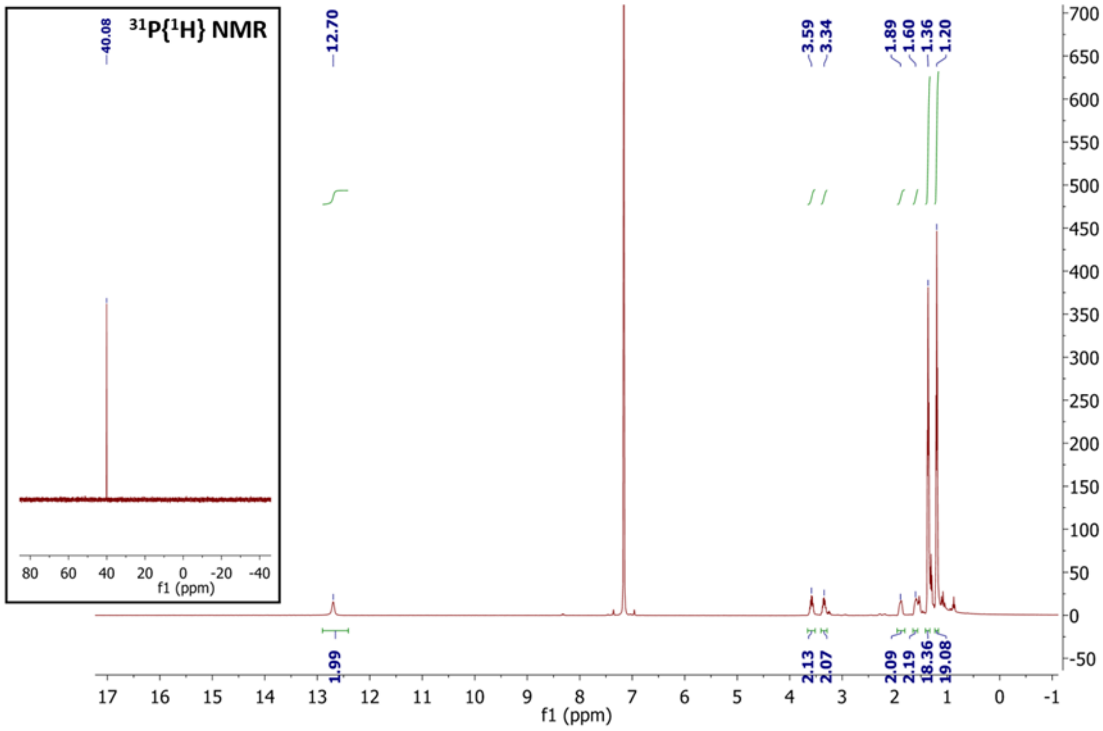

Figure S1. ^{1}H (400 MHz, C_6D_6) and $^{31}P\{^1H\}$ NMR spectra (162 MHz, C_6D_6, inset) of **3**.

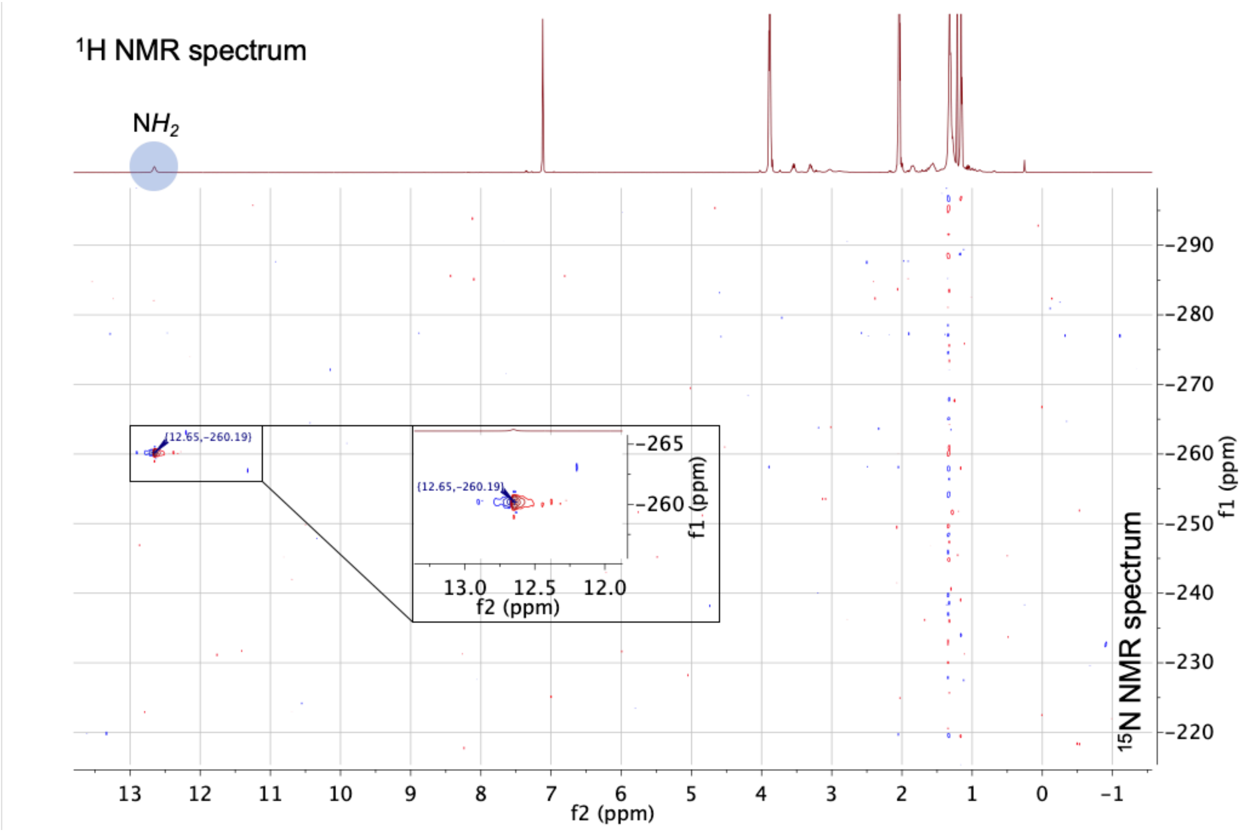

Figure S2. ^{1}H–^{15}N HSQC spectrum (400 MHz/51 MHz, C_6D_6) of **3** showing a ^{15}N cross-peak at δ –260 ppm corresponding to the 2 H resonance at δ 12.7 ppm.

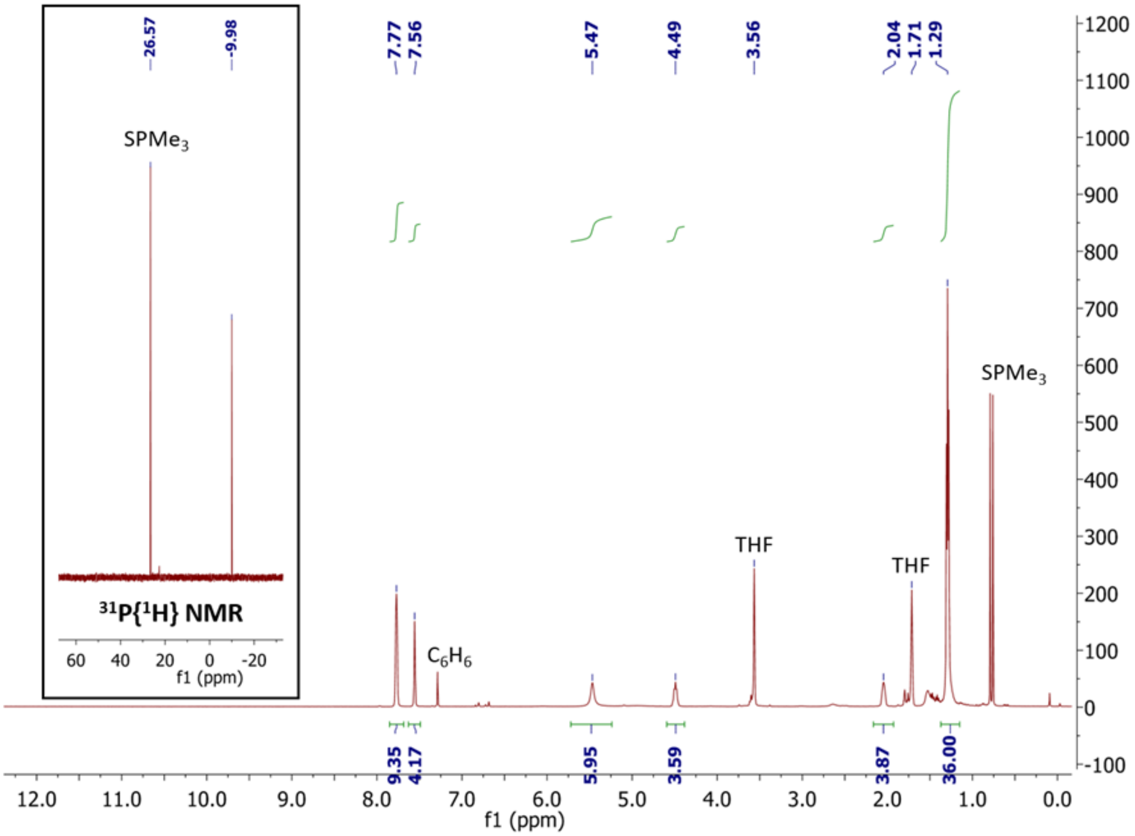

Figure S3. ^{1}H (400 MHz, THF-d_8) and $^{31}P\{^{1}H\}$ NMR spectra (162 MHz, THF-d_8, inset) of **4**.

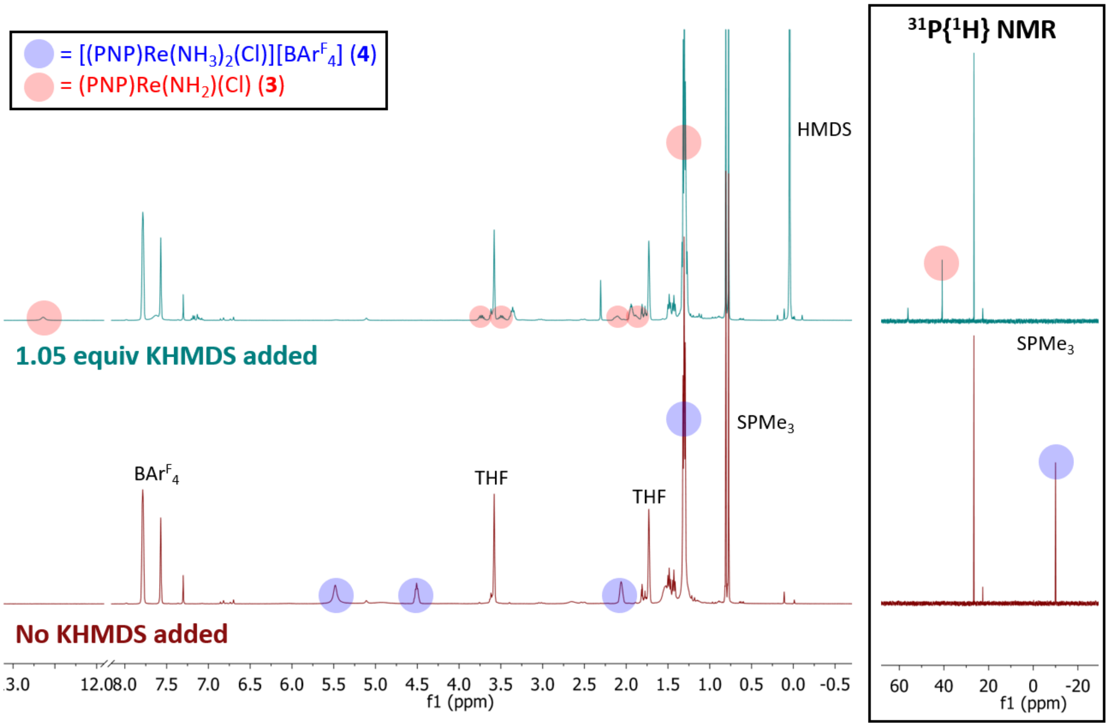

Figure S4. ^{1}H (400 MHz, THF-d_8) and $^{31}P\{^{1}H\}$ (162 MHz, THF-d_8) NMR spectra of **4** in THF-d_8 before (bottom, maroon) and after (top, teal) the addition of 1.05 equiv KHMDS. Complex **3** is formed in 61% spectroscopic yield vs. $SPMe_3$ in a capillary.

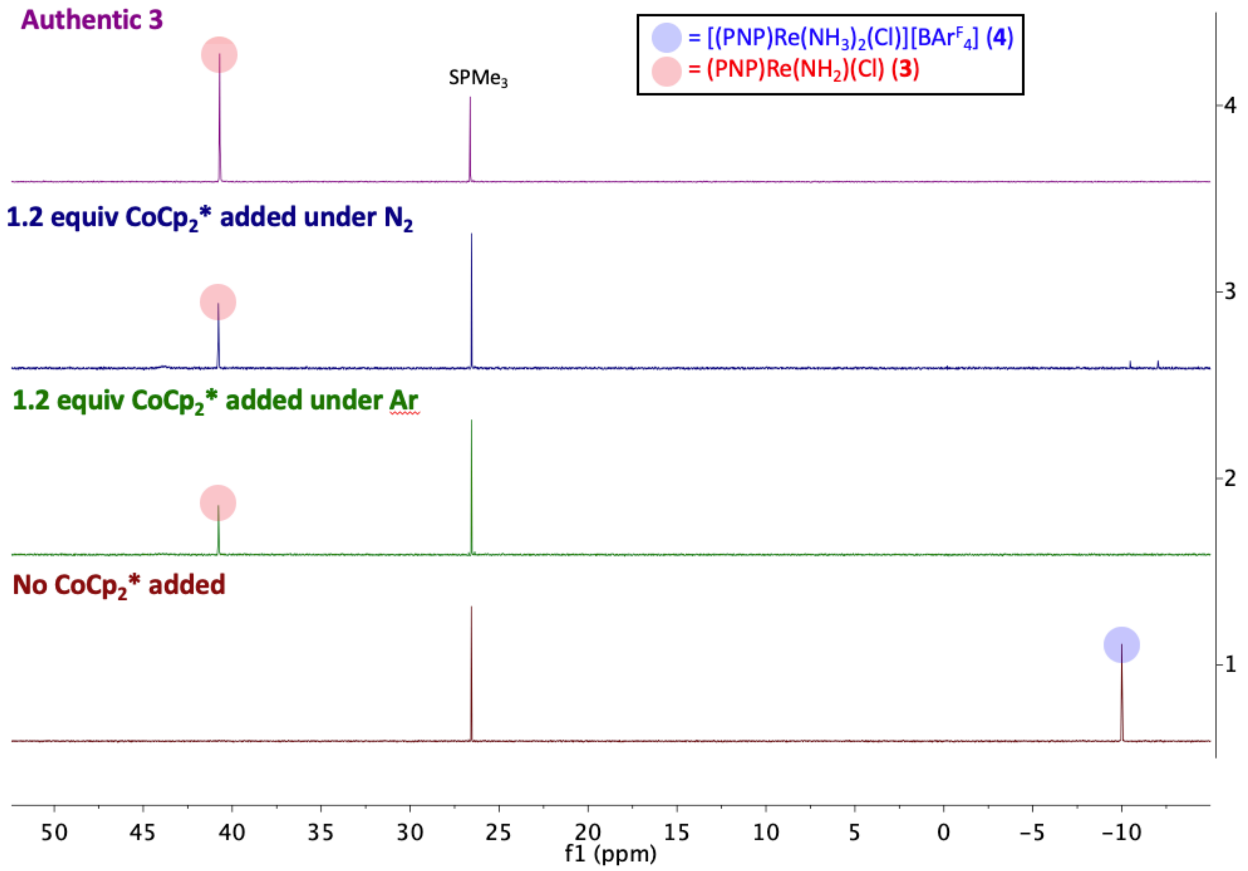

Figure S5A. $^{31}P\{^1H\}$ NMR spectra (162 MHz, THF-d_8) of **4** in THF-d_8 prior to (maroon) and after addition of 1.2 equiv $CoCp_2$*. Under Ar, the reaction gave **3** in 60% spectroscopic yield (green). Under N_2, the reaction gave **3** in 60% spectroscopic yield, along with other unidentified minor products (blue). Spectroscopic yields measured vs. $SPMe_3$ in a capillary. For comparison, a spectrum of authentically prepared **3** is included (purple).

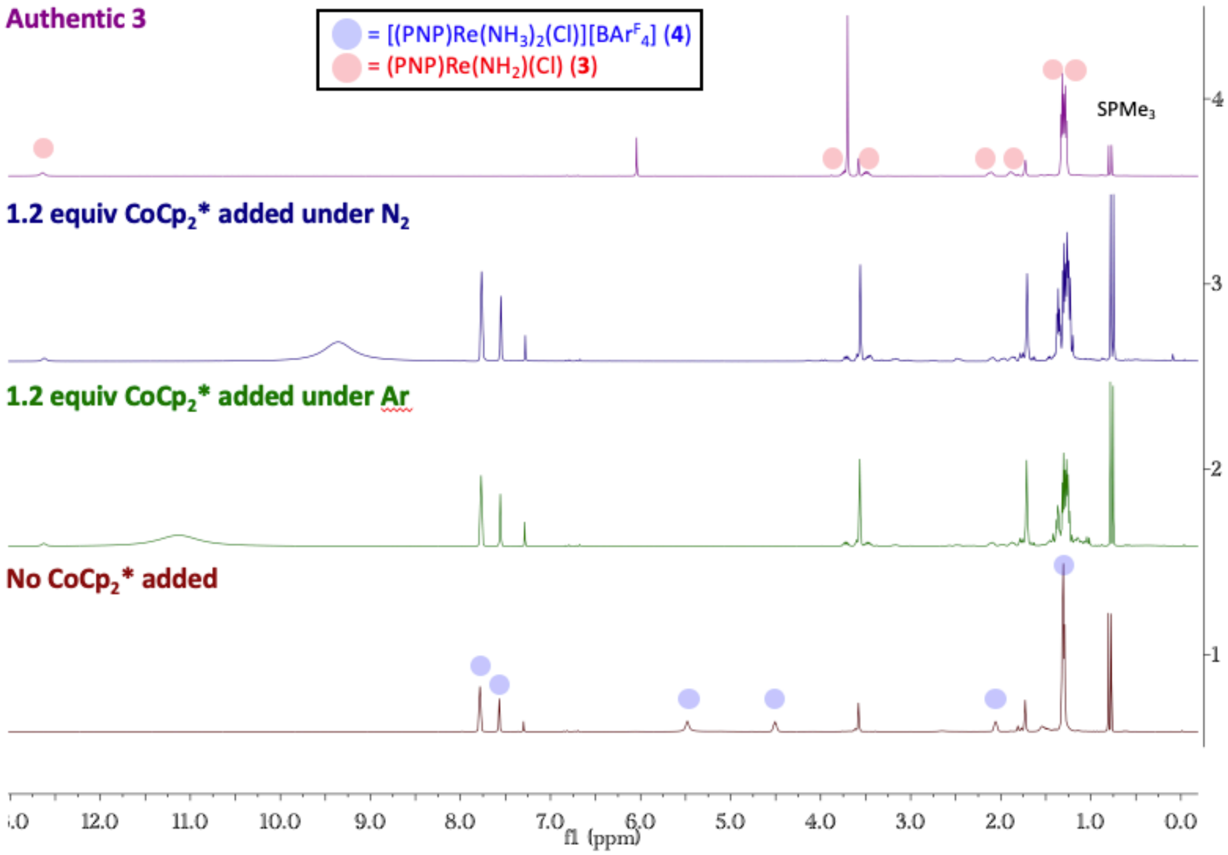

Figure S5B. ^{1}H NMR spectra (400 MHz, THF-d_8) of **4** in THF-d_8 prior to (maroon) and after addition of 1.2 equiv $CoCp_2$*. Under Ar, the reaction gave **3** in 60% spectroscopic yield (green). Under N_2, the reaction gave **3** in 60% spectroscopic yield, along with other unidentified minor products (blue). Spectroscopic yields measured vs. $SPMe_3$ in a capillary. For comparison, a spectrum of authentically prepared **3** is included (purple). The broad peaks at 9-11 ppm are presumed to be from Cp^*_2Co and $Cp^*_2Co^+$ in fast exchange, and integrate to 2.9 Co per mol **3** in the N_2 spectrum and 2.5 Co per mol **3** in the Ar spectrum.

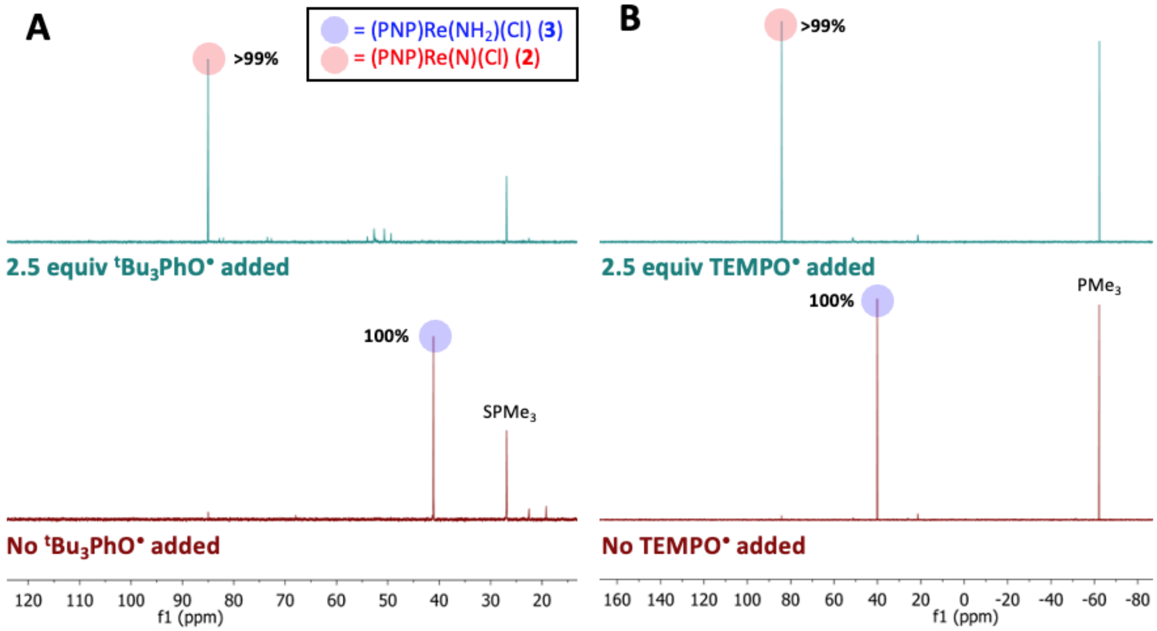

Figure S6. ${}^{31}P\{{}^{1}H\}$ NMR spectra (202 MHz, THF-d_8 or 162 MHz, C_6D_6) of the reaction of **3** with organic H-atom abstracting (HAA) reagents. **A:** Solution of **3** in THF-d_8 before (bottom, maroon) and after (top, teal) addition of 2.5 equiv ${}^{t}Bu_3PhO^{\bullet}$ at ambient temperature, showing conversion to nitride complex **2** within minutes. This initial experiment had small impurities, which were not present in the sample of **3** used later for calorimetry experiments. **B:** Solution of **3** in C_6D_6 before (bottom, maroon) and after (top, teal) addition of 2.5 equiv TEMPO$^{\bullet}$ at ambient temperature, showing conversion to nitride complex **2** within minutes. Spectroscopic yields were calculated vs. an internal standard of $SPMe_3$ or PMe_3 in a capillary.

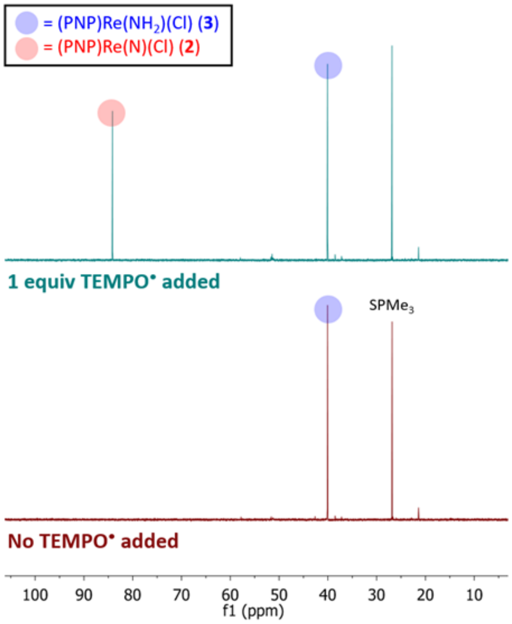

Figure S7. ${}^{31}P\{{}^{1}H\}$ NMR spectra (202 MHz, THF-d_8 or 162 MHz, C_6D_6) of a solution of **3** in C_6D_6 before (bottom, maroon) and after (top, teal) addition of 1 equiv TEMPO$^{\bullet}$, resulting in formation of **2**.

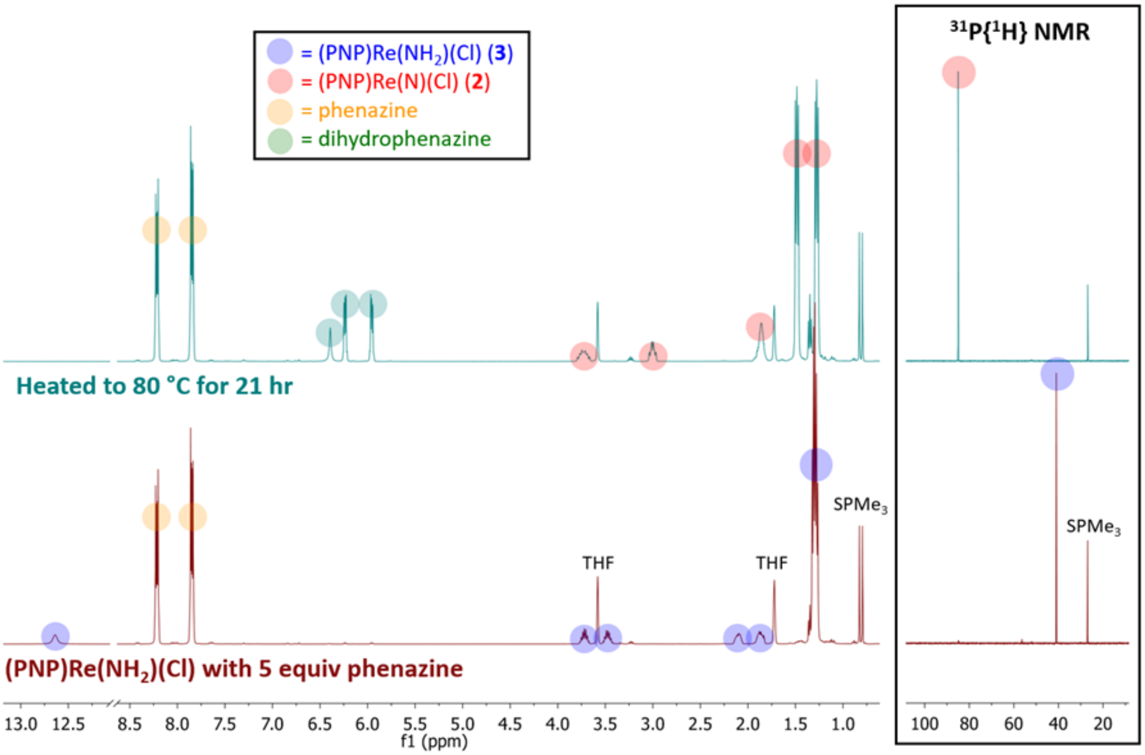

Figure S8. ^{1}H (400 MHz, THF-d_8) and $^{31}P\{^{1}H\}$ (162 MHz, THF-d_8) NMR spectra of **3** and 5 equiv phenazine in THF-d_8 before (bottom, maroon) and after (top, teal) heating at 80 °C for 21 h. Complex **2** is formed in 99% spectroscopic yield vs. $SPMe_3$ in a capillary.

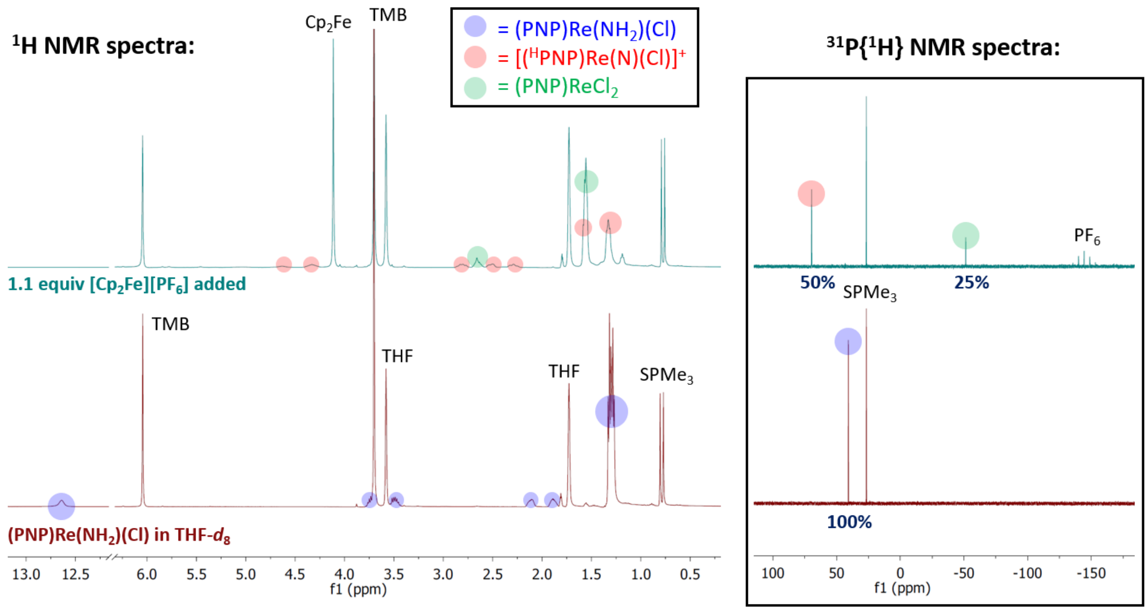

Figure S9. ^{1}H NMR (400 MHz, THF-d_8) and $^{31}P\{^{1}H\}$ NMR (162 MHz, THF-d_8) spectra of **3** before (bottom, maroon) and after the addition of 1.1 equiv $[Cp_2Fe][PF_6]$ (top, teal), showing the formation of **5** and **1**. Spectroscopic yields are reported vs. TMB as an internal standard and a $SPMe_3$ capillary.

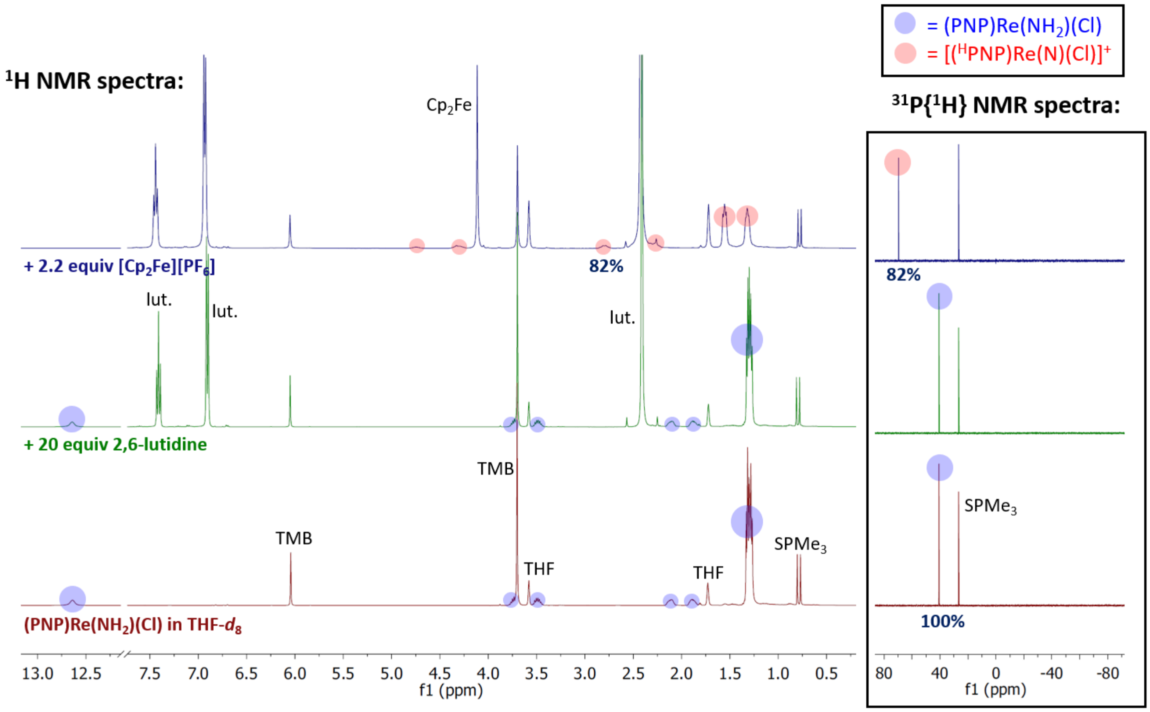

Figure S10. ^{1}H NMR (400 MHz, THF-d_8) and $^{31}P\{^{1}H\}$ NMR (162 MHz, THF-d_8) spectra of **3** (bottom, maroon) after the addition of 20 equiv 2,6-lutidine (middle, green), then after the addition of 2.2 equiv $[Cp_2Fe][PF_6]$ (top, green). **3** shows no reaction with 2,6-lutidine, but reacts with 2.2 equiv $[Cp_2Fe][PF_6]$ to give **5** in 82% yield, accompanied by formation of a brown precipitate. No **1** is formed in the reaction. Spectroscopic yields are reported vs. TMB as an internal standard and a $SPMe_3$ capillary.

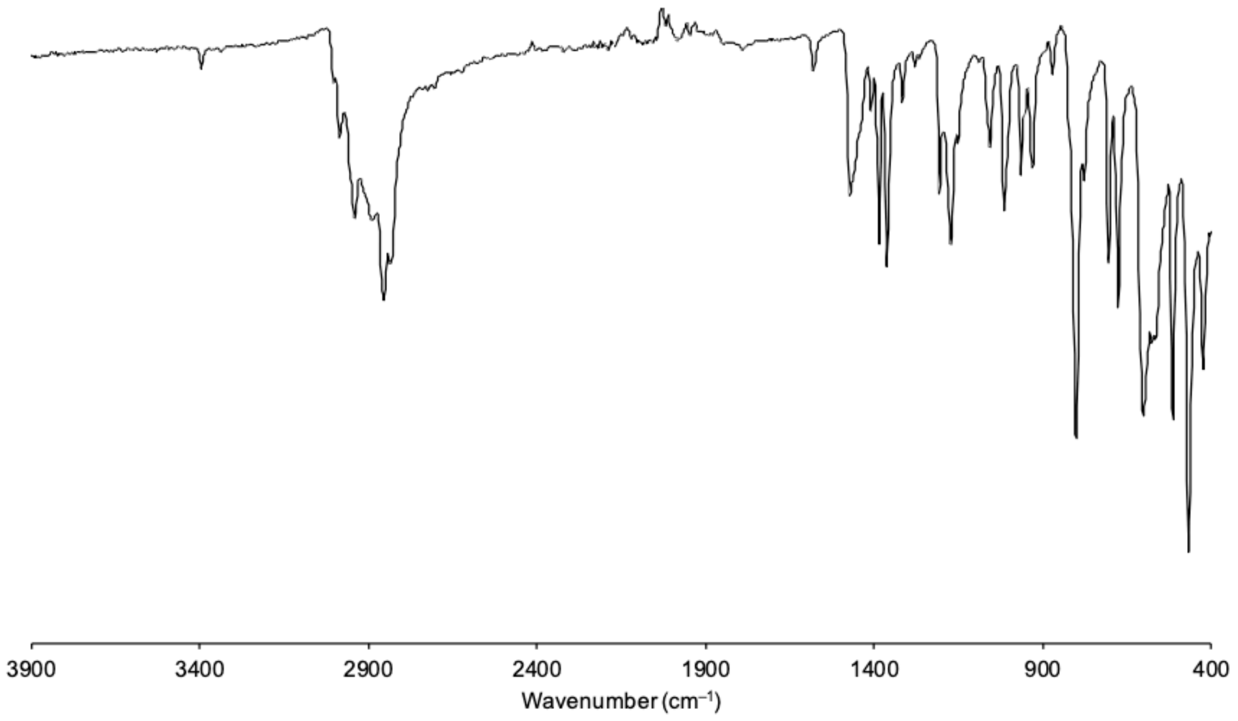

Figure S11. FTIR (solid) spectrum of **3**.

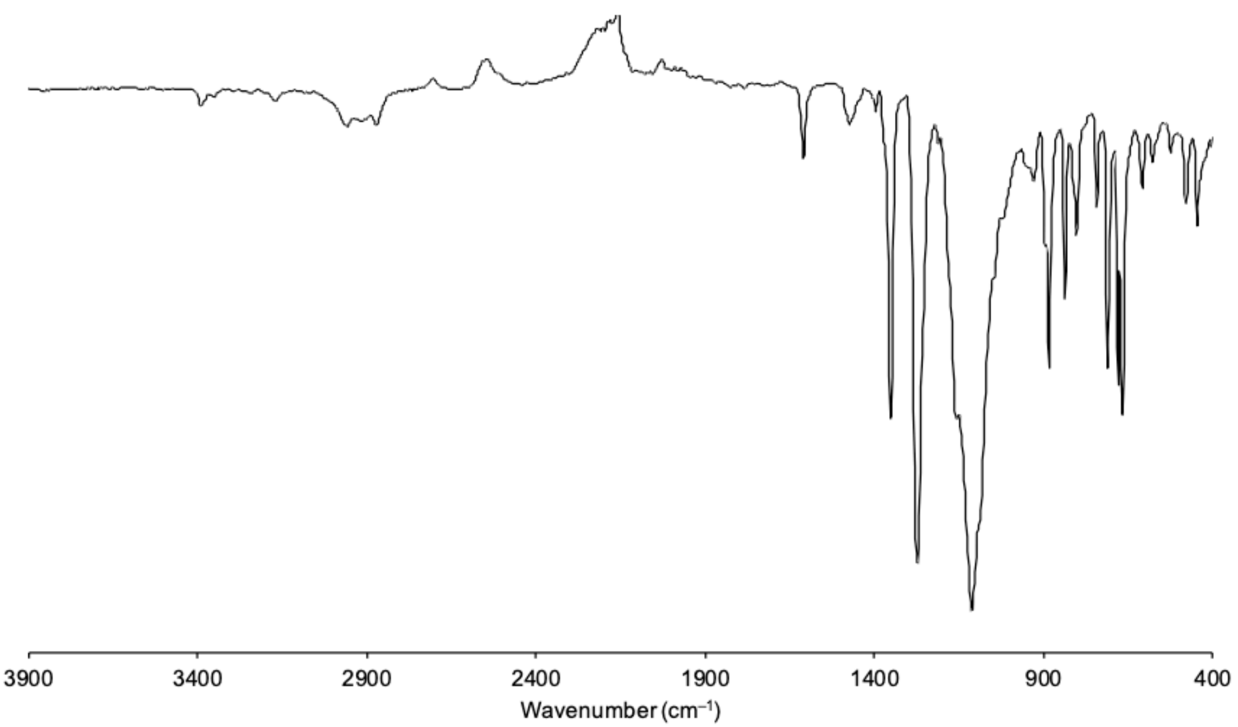

Figure S12. FTIR (solid) spectrum of **4**.

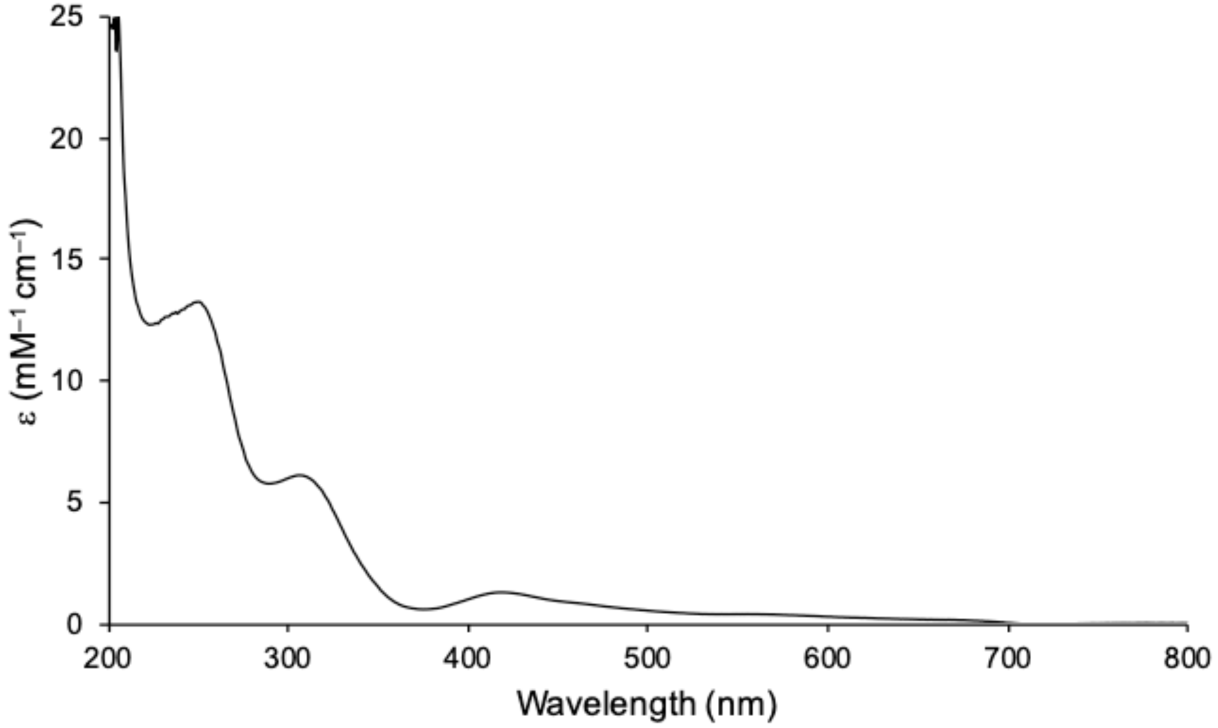

Figure S13. UV-Vis spectrum of a 1.57 mM solution of **3** in THF using a 1 mm path length cell.

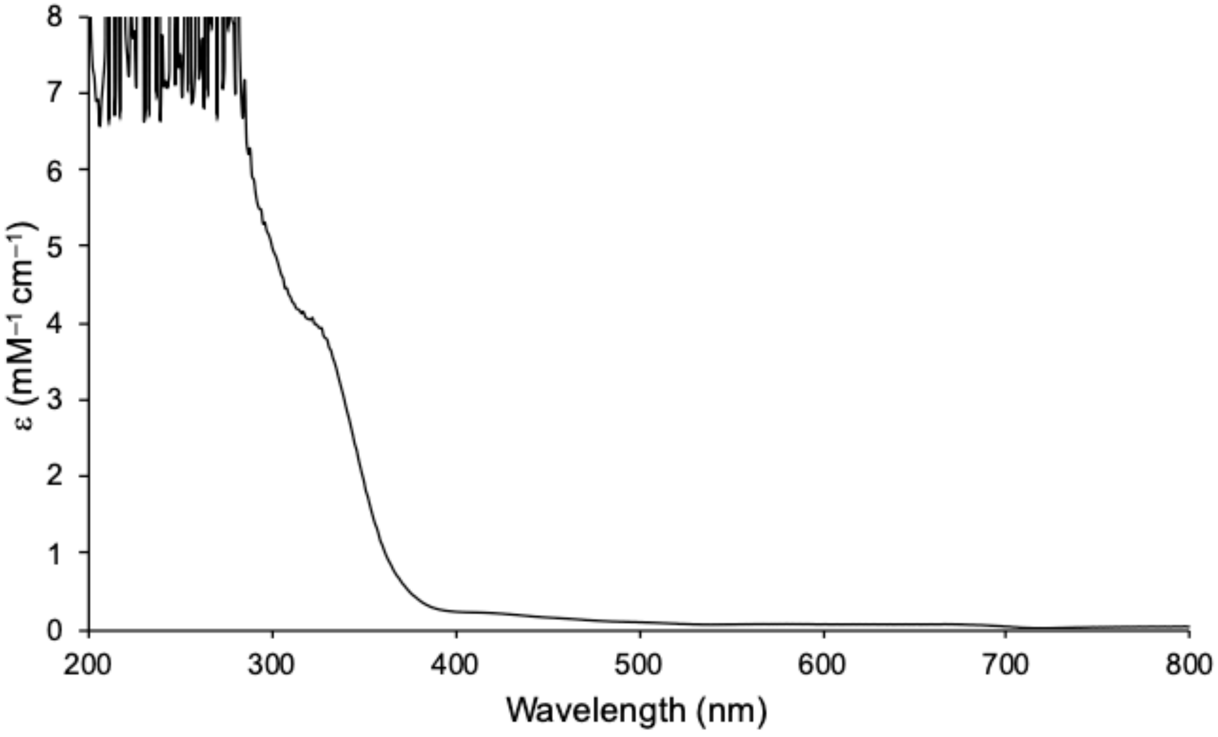

Figure S14. UV-Vis spectrum of a 5.68 mM solution of **4** in THF using a 1 mm path length cell.

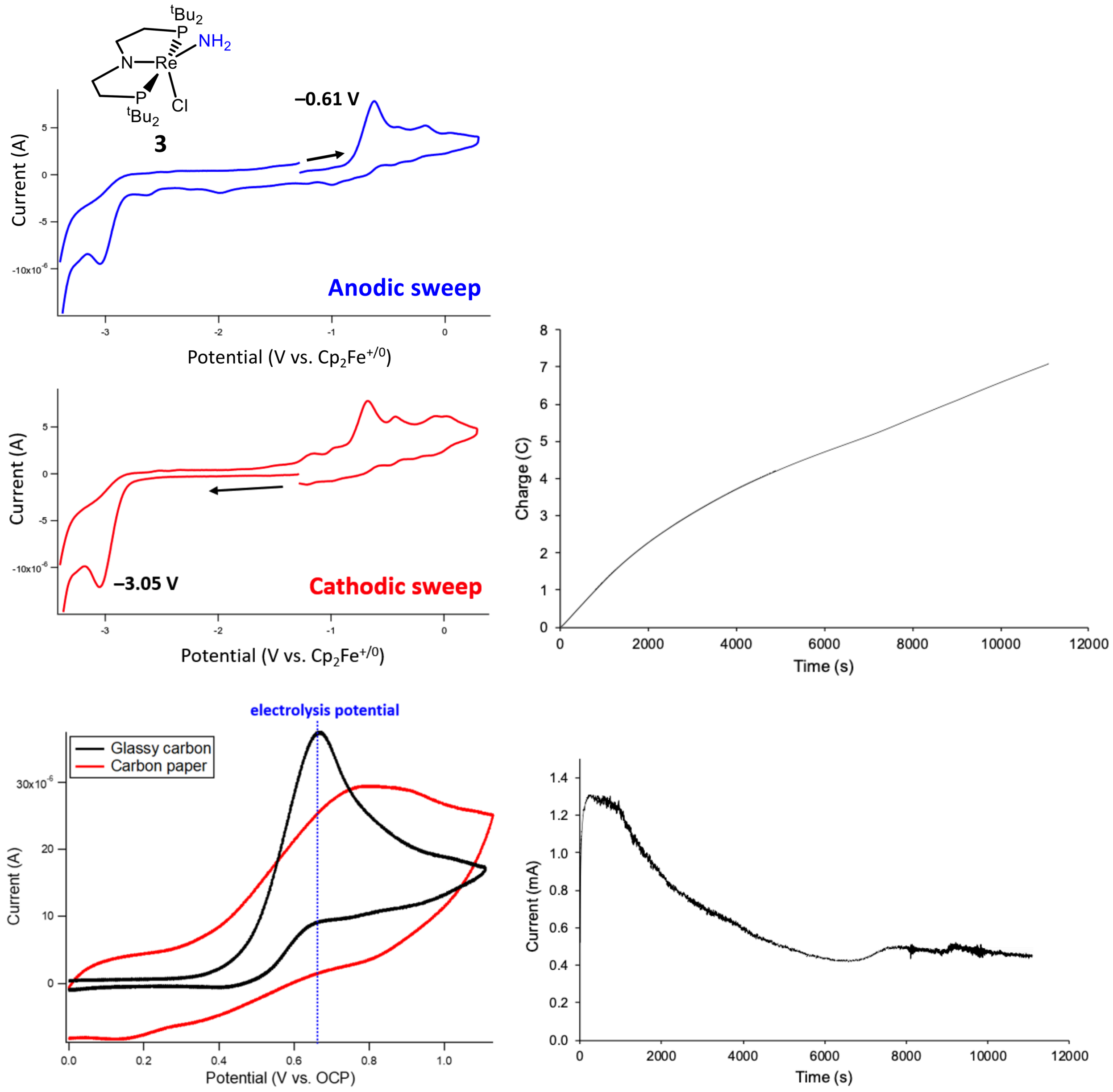

Figure S15. Top: Cyclic voltammogram of **3** in 0.2 M tetrabutylammonium hexafluorophosphate solution in THF under N_2. CVs measured at 100 mV/s with a glassy carbon disc working electrode, Pt wire auxiliary electrode, and Ag wire psuedoreference. Figure 4 in the text shows that the anodic response is pseudoreversible at faster scan rates. Potentials referenced to $Cp_2Fe^{+/0}$ after the experiments. **Bottom left:** Comparison of cyclic voltammograms of **3** (1.7 mM) prior to electrolysis, measured in 0.2 M tetrabutyl-ammonium hexafluorophosphate solution in THF containing excess 2,6-lutidine (34 mM) under Ar, using a glassy carbon (black) or carbon paper (red) working electrode, Pt mesh counter electrode, and Ag wire pseudoreference electrode. The electrolysis potential (+0.66 V vs. open circuit potential) is noted in blue. **Right:** Charge passed vs. time, and current vs. time, during the electrolysis of **3**. The electrolysis was stopped after 2.2 equiv of charge per Re was passed. The continued increase of charge passed is likely due to degradation and contributes to a low yield.

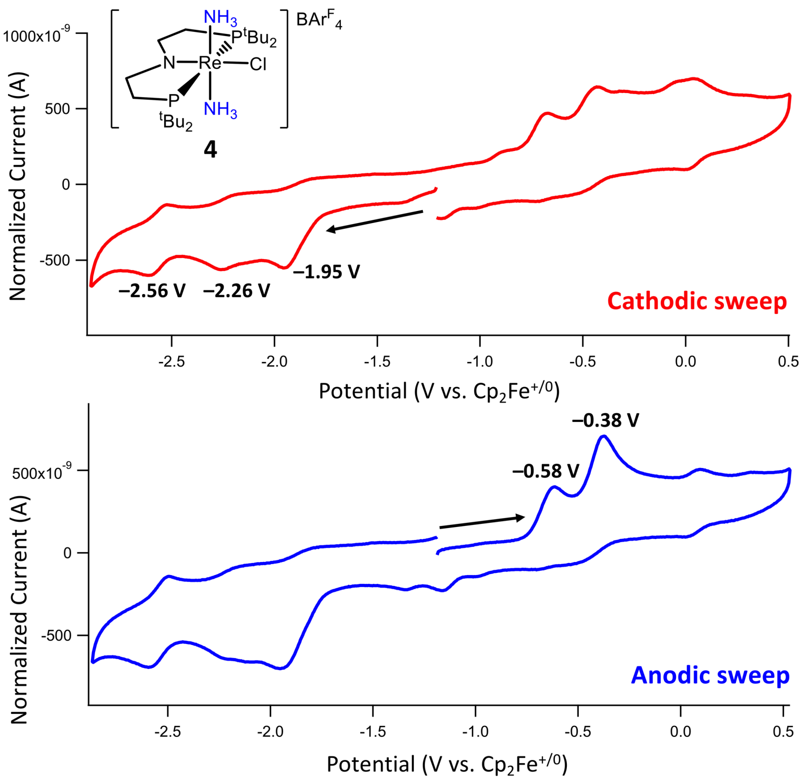

Figure S16. Cyclic voltammograms (CVs) of 0.4 mM **4** in 0.2 M tetrabutylammonium hexafluorophosphate solution in THF under Ar, demonstrating irreversible electrochemical events sweeping in the cathodic (reductive) direction (top, red) and the anodic (oxidative) direction (bottom, blue). CVs measured at 100 mV/s with a glassy carbon disc working electrode, Pt wire auxiliary electrode, and Ag wire pseudoreference. Potentials referenced to $Cp_2Fe^{+/0}$ after the experiments.

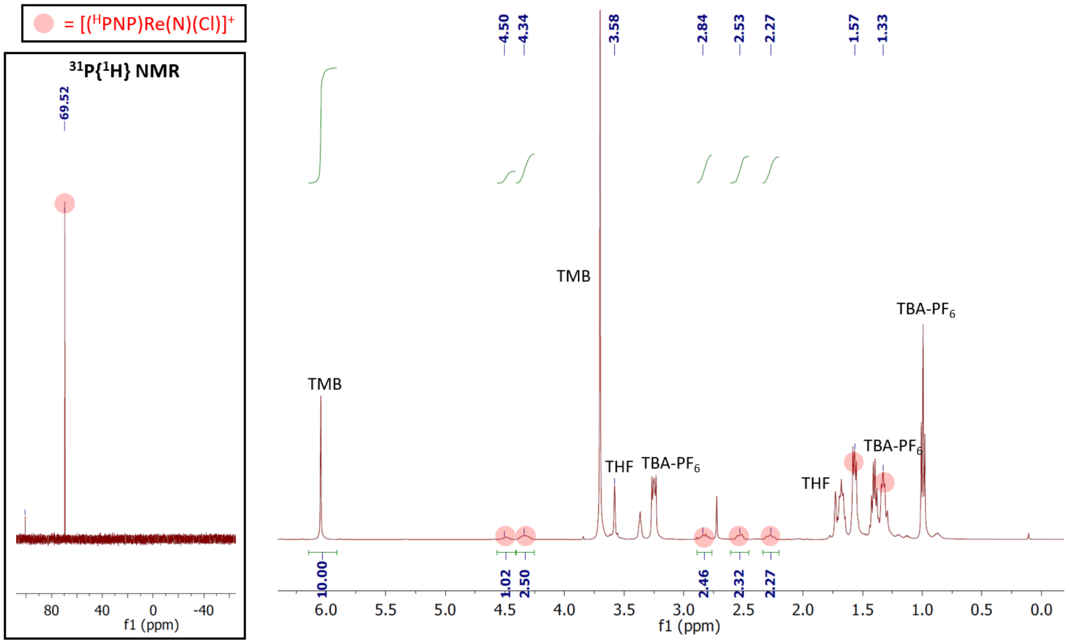

Figure S17. ^{1}H NMR (400 MHz, THF-d_8) and $^{31}P\{^{1}H\}$ NMR (162 MHz, THF-d_8) spectra of the THF-soluble products isolated from 2.2e^{-} electrolysis of **3** in the presence of 20 equiv 2,6-lutidine, showing formation of **5** in 69% yield. No **1** was isolated from the electrolysis. Yield reported vs. a TMB internal standard.

Calorimetric titration of (PNP)Re(NH_2)(Cl) (3) with $^tBu_3PhO^•$: Isothermal Titration Calorimetry (ITC) was performed in a NanoITC device by TA Instruments with a 24k gold cell and a sample volume of 1 mL operated in overfill mode. 2,4,6-tri-*tert*-butylphenoxyl radical ($^tBu_3PhO^•$) was used as the titrant. We used ITCRun Version 3.4.6.0 and NanoAnalyze Version 3.7.5.

The experimental conditions for the titration of (PNP)Re(NH_2)(Cl) (**3**) with $^tBu_3PhO^•$ in THF are summarized in **Table S1**. Three separate runs were performed, and all measurements were taken at ambient temperature (298 K).

Table S1. Reaction details for isothermal titration calorimetry experiments.

	First Run	Second Run	Third Run
Concentration and volume of **3** (mM/µL)	0.341 / 950	0.341 / 950	0.341 / 950
Concentration and volume of TBP (mM/µL)	5.28 / 250	5.05 / 180	5.05 / 180
Injection Steps x Injected Volume (µL)	33 x 7.5	24 x 7.5	24 x 7.5
Waiting time between two additions (s)	250	250	250
Stirring rate (rpm)	350	350	350
Equilibration time before start of experiment (s)	1885	1453	1525

Figure S18 shows a representative thermogram and integrated titration curve for the titration of **3** with $^tBu_3PhO^•$ in THF. Greyed out points are not taken into account in the fitting process. The first point is generally neglected due to the dilution effect within the pre-experiment equilibration time. The mean heat value was determined for the titration steps before the equivalence point.

Table S2. Summarized results from the three independent isothermal calorimetic titrations.

Run	Mean Heat [µJ]	Consumed moles per injection [nmol]	Enthalpy [kJ/mol]
1	-4176 ± 27	19.62	-213 ± 1
2	-3957 ± 40	18.76	-211 ± 2
3	-4017 ± 39	18.76	-214 ± 2

$$\textbf{Mean Enthalpy} = -213 \pm 2\,\frac{\text{kJ}}{\text{mol}} = -50.9 \pm 0.5\,\frac{\text{kcal}}{\text{mol}}$$

From the experimental reaction enthalpy of the reaction of amide **3** with TBP (2 equiv.) to nitride **2** in THF at 298 K (–50.9 ± 0.5 kcal/mol), the average $BDE_{N–H}$ of **3** was calculated to be 55.4 ± 1 kcal/mol according to the following equations. The $BDE_{O–H}$ of $^tBu_3PhO^•$ in THF (80.8 ± 0.3 kcal/mol) was estimated from the experimental $BDFE_{O–H}$,[3] assuming negligible differential entropies of solution of $^tBu_3PhO^•$ and $^tBu_3PhOH$ and using $TS^0(H\cdot) = 6.4$ kcal mol^{-1} as entropy of solvation of the hydrogen atom based on the entropy of solvation of H_2.[6]

$$\text{BDE}^{\text{THF}}_{[\text{Re}]\text{N(H)}-\text{H}} = \text{BDE}^{\text{THF}}_{\text{TBP}} - \frac{\Delta H}{2}$$

$$\text{BDE}^{\text{THF}}_{[\text{Re}]\text{N(H)}-\text{H}} = \left(80.8 - \frac{50.9}{2}\right)\frac{\text{kcal}}{\text{mol}} = \mathbf{55.4 \pm 1\,\frac{kcal}{mol}}$$

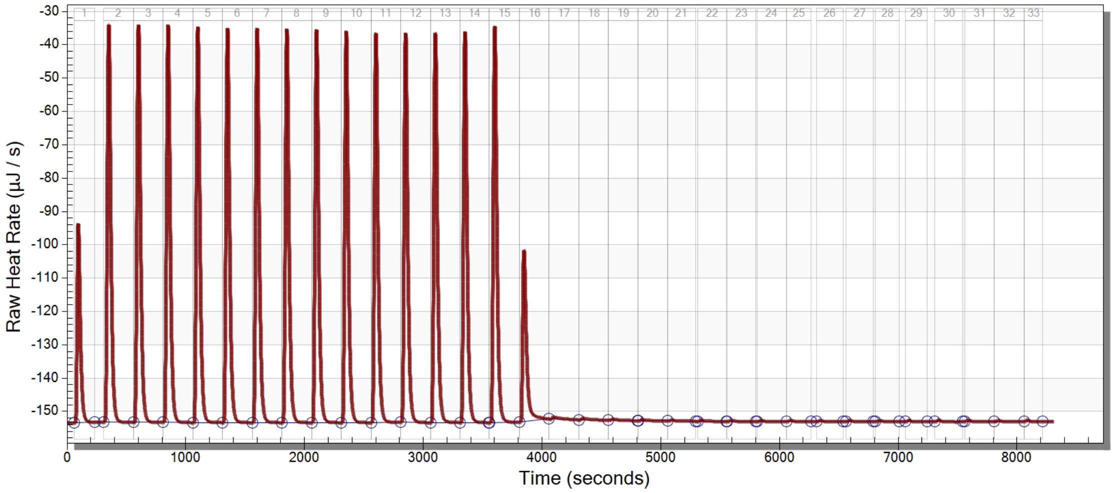
Raw Heat Rate (µJ / s)
Time (seconds)

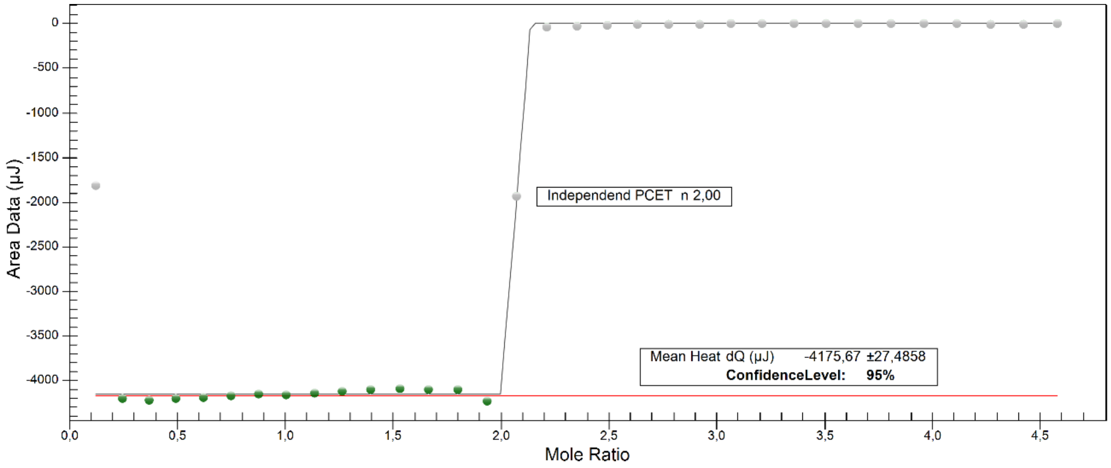
Area Data (µJ)
Mole Ratio
Independend PCET n 2,00
Mean Heat dQ (µJ) -4175,67 ±27,4858
ConfidenceLevel: 95%

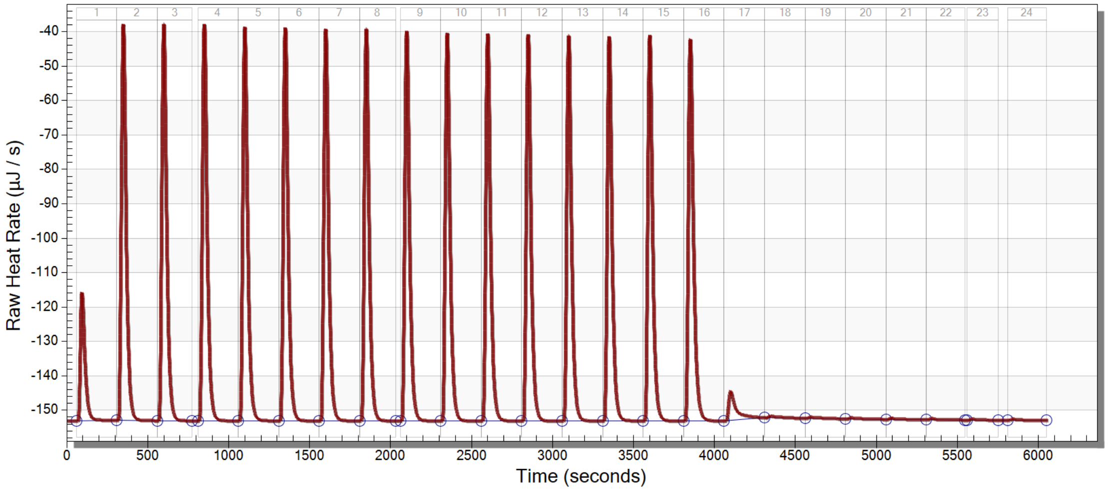
Raw Heat Rate (µJ / s)
Time (seconds)

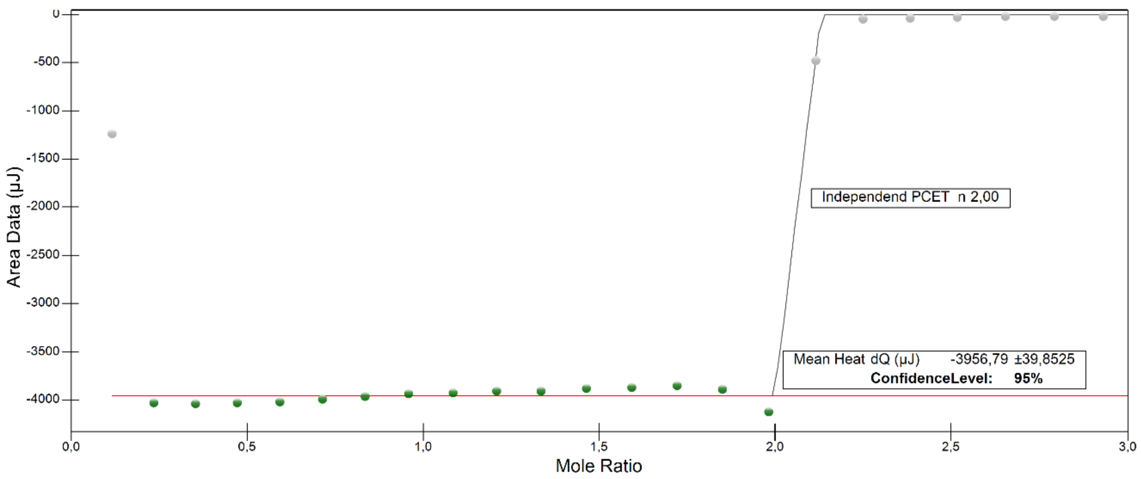

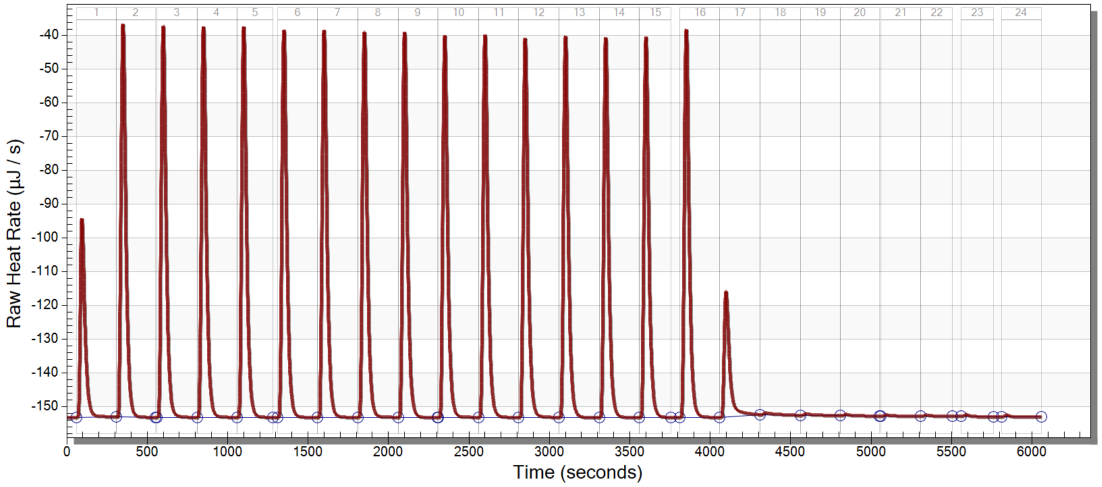

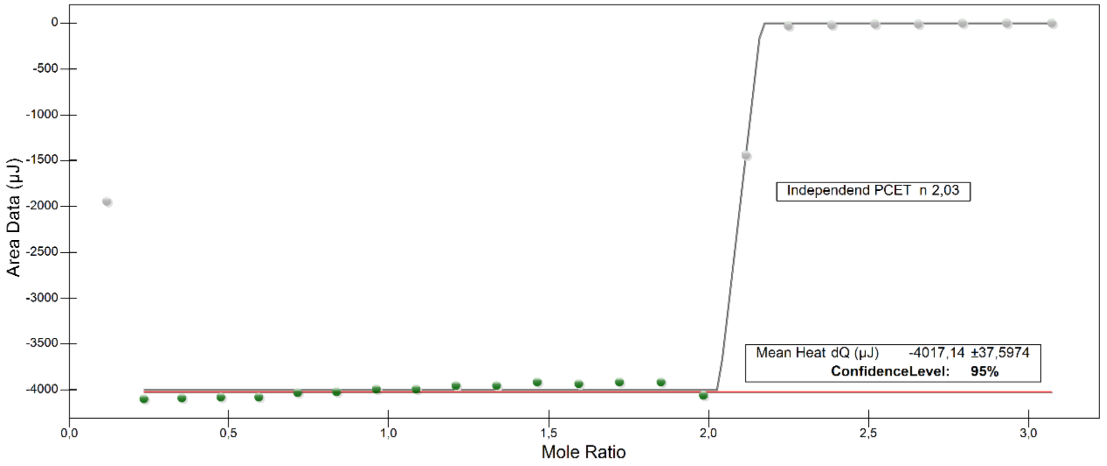

Figure S18. Top: Integrated heat titration curve at 298 K for the reaction of **3** with up to 4.5 equiv $^{t}Bu_3PhO^{\bullet}$ in THF, showing equivalence at 2.0 equiv of oxidant. **Bottom:** Thermogram of the corresponding titration. The reaction exhibits a mean exotherm of –50.9 kcal/mol over three runs.

Computational details

Density Functional Theory (DFT) calculations were performed using Gaussian 09 (revision D.01).[7] Geometries were optimized using the hybrid functional B3LYP, with basis set def2-TZVP used for all atoms and an ultrafine integration grid. Unless otherwise noted, all calculations include the GD3BJ version of Grimme's dispersion correction and a conductor-like polarizable continuum model (CPCM) for THF solvent. Frequency calculations were performed to confirm that the optimized structures were minima; the optimized structures for the complexes reported showed no negative frequencies.

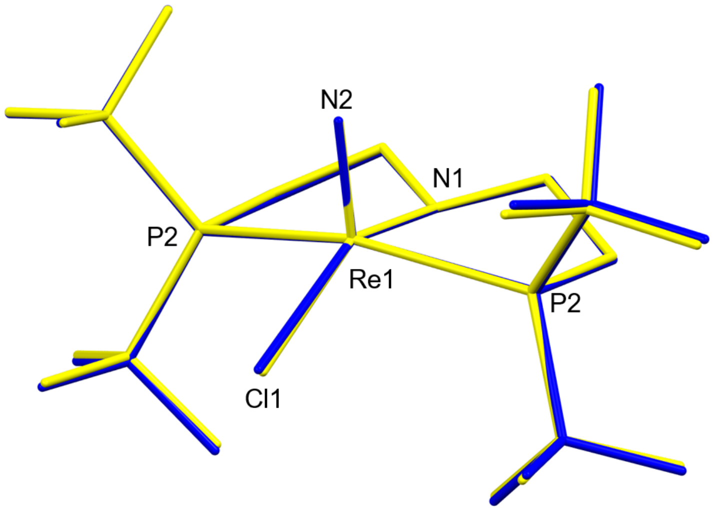

Figure S19. Overlay of crystal structure (yellow) and calculated structure (blue) of **2**. Hydrogen atoms are omitted for clarity.

Table S3. Calculated structural parameters of **2**.

Bond (Å) / Angle (°)	Experimental	Calculated
Re – N1	2.033(6)	1.029
Re – N2	1.643(6)	1.663
Re – Cl1	2.441(2)	2.517
Re – P (avg)	2.439	2.464
N1 – Re - N2	105.8(3)	106.0
N1 – Re – Cl1	147.7(2)	150.3
N2 – Re – Cl1	106.5(2)	103.8

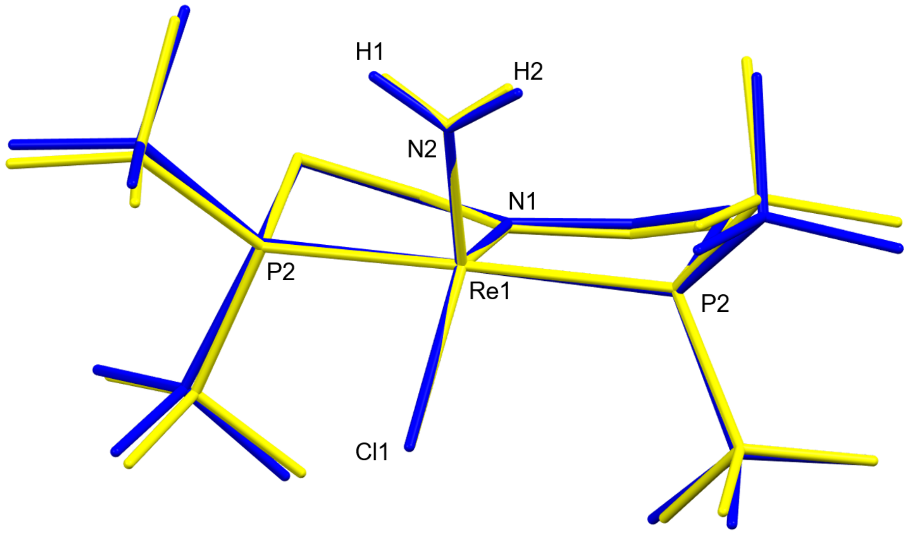

Figure S20. Overlay of crystal structure (yellow) and calculated structure (blue) of **3**. Hydrogen atoms (other than amide hydrogens) are omitted for clarity.

Table S4. Calculated structural parameters of **3**.

Bond (Å) / Angle (°)	Experimental	Calculated
Re – N1	1.936(3)	1.952
Re – N2	1.959(3)	1.930
Re – Cl1	2.384(1)	2.440
Re – P (avg)	2.390	2.407
N1 – Re - N2	115.5(1)	114.7
N1 – Re – Cl1	135.6(1)	138.3
N2 – Re – Cl1	108.8(1)	107.1

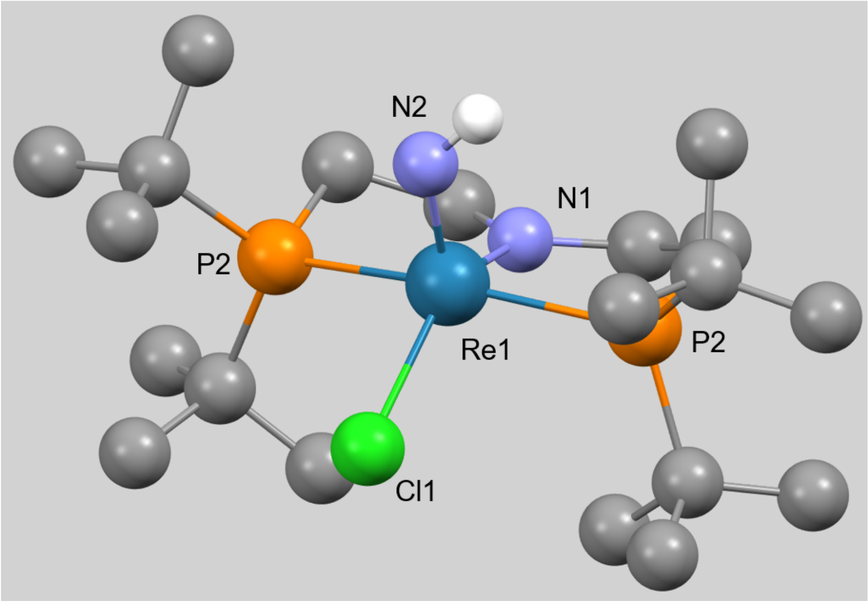

Figure S21. Calculated structure of **LRe=NH**. Hydrogen atoms (other than imide hydrogen) are omitted for clarity.

**Bond (Å) / Angle (°)**	**Calculated**
Re – N1	1.988
Re – N2	1.796
Re – Cl1	2.468
Re – P (avg)	2.440
N1 – Re – N2	112.2
N1 – Re – Cl1	141.8
N2 – Re – Cl1	105.9

Table S9. Calculated reaction enthalpies of H-atom abstraction from **3** and **LRe=NH** with $^{t}Bu_3PhO^{\bullet}$ in THF and resulting $BDE_{N\text{–}H}$ values. Referenced vs. $^{t}Bu_3PhOH$ ($BDE_{O\text{–}H}$ = 80.8 kcal/mol).[3]

Reaction	Reaction Enthalpy (kcal/mol)	BDE (kcal/mol)
**3** + $tBu_3PhO^{\bullet}$ -> Re=NH + tBu_3PhOH	-4.1	76.7
Re=NH + $tBu_3PhO^{\bullet}$ -> **2** + tBu_3PhOH	-43.2	37.6
Average		57.2

Table S10. Calculated reaction free energies of H-atom abstraction from **3** and **LRe=NH** with $^{t}Bu_3PhO^{\bullet}$ in THF and resulting $BDFE_{N\text{–}H}$ values. Referenced vs. $^{t}Bu_3PhOH$ ($BDFE_{O\text{–}H}$ = 74.4 kcal/mol).[3]

Reaction	Reaction Free Energy (kcal/mol)	BDFE (kcal/mol)
**3** + $tBu_3PhO^{\bullet}$ -> Re=NH + tBu_3PhOH	-5.0	69.4
Re=NH + $tBu_3PhO^{\bullet}$ -> **2** + tBu_3PhOH	-41.7	32.7
Average		51.0

Redox potential computation:

The redox potential of the **2**/**3** couple was computed using the structurally related $[Re^{VI}(N)Cl(PNP)]^{+}$ / $[Re^{V}(N)Cl(PNP)]$ redox couple ($E^{DFT}{}_{ref}$) as reference, where a redox potential of E = -0.086 V was reported:[8]

$$E = E^{DFT}(\mathbf{2}/\mathbf{3}) - E^{DFT}{}_{ref} + (-0.086\ \text{V})$$

Coordinates for calculated structures:

(PNP)Re(N)(Cl) (**2**):

```
Re 0.00000 -0.25322 -0.20980
Cl 0.00000 1.50952 -2.00649
P 2.40299 -0.01156 0.27720
P -2.40299 -0.01156 0.27720
N 0.00000 -1.68393 -1.05823
N 0.00000 -0.76838 1.75296
C 2.44084 -0.53554 2.04396
H 3.35598 -1.07320 2.29092
H 2.42258 0.37166 2.64616
C 1.19495 -1.35356 2.35804
H 1.33061 -2.39406 2.02764
H 1.07168 -1.39307 3.44751
C -1.19495 -1.35356 2.35804
H -1.07168 -1.39308 3.44751
H -1.33061 -2.39406 2.02764
C -2.44084 -0.53554 2.04396
H -2.42258 0.37166 2.64616
H -3.35598 -1.07320 2.29092
```

```
C 3.50468 -1.21716 -0.66514
C 3.25772 -1.06067 -2.17183
H 2.21363 -1.24629 -2.41575
H 3.87234 -1.79032 -2.70506
H 3.52416 -0.07080 -2.53541
C 4.99401 -1.02982 -0.35625
H 5.38060 -0.08821 -0.73980
H 5.55227 -1.83471 -0.84070
H 5.20259 -1.08320 0.71264
C 3.10037 -2.64061 -0.25061
H 3.33392 -2.84325 0.79424
H 3.66880 -3.34814 -0.85898
H 2.04101 -2.82251 -0.41843
C 3.12640 1.73249 0.32130
C 3.47823 2.20993 -1.09236
H 4.34534 1.68970 -1.49609
H 3.72492 3.27419 -1.05140
H 2.63751 2.08327 -1.77271
C 4.35605 1.85401 1.23260
H 4.13589 1.57852 2.26322
H 4.67696 2.89889 1.23687
H 5.19468 1.25412 0.89148
C 2.01631 2.64146 0.88032
H 1.18882 2.72731 0.17975
H 2.42976 3.64022 1.04007
H 1.62843 2.28790 1.83658
C -3.50468 -1.21716 -0.66514
C -3.25772 -1.06067 -2.17183
H -3.52417 -0.07079 -2.53541
H -3.87235 -1.79032 -2.70506
H -2.21364 -1.24629 -2.41575
C -3.10037 -2.64061 -0.25061
H -2.04101 -2.82251 -0.41843
H -3.66880 -3.34814 -0.85898
H -3.33391 -2.84325 0.79423
C -4.99401 -1.02982 -0.35624
H -5.20259 -1.08320 0.71264
H -5.55227 -1.83471 -0.84070
H -5.38061 -0.08821 -0.73980
C -3.12640 1.73249 0.32130
C -3.47823 2.20993 -1.09236
H -2.63752 2.08327 -1.77271
H -4.34535 1.68970 -1.49609
C -2.01631 2.64146 0.88032
H -1.62843 2.28790 1.83658
H -2.42976 3.64022 1.04007
H -1.18883 2.72731 0.17975
C -4.35605 1.85401 1.23260
H -5.19468 1.25412 0.89149
H -4.67696 2.89889 1.23687
H -4.13589 1.57852 2.26322
H -3.72492 3.27419 -1.05139
```

(PNP)Re(NH)(Cl) (**Re=NH**):

```
Re 0.00537 -0.34364 -0.01187
P -2.41070 -0.01531 0.24666
P 2.39527 0.00740 0.24563
N -0.00342 0.43338 1.81747
C -2.45658 0.06275 2.08349
H -3.35955 0.51783 2.48735
H -2.42887 -0.97201 2.41896
C -1.20570 0.76739 2.59325
```

H -1.35960 1.85512 2.60731
H -1.04736 0.47846 3.63948
C 1.18551 0.85805 2.56805
H 1.04971 0.60995 3.62758
H 1.28678 1.95096 2.52962
C 2.46266 0.18947 2.07671
H 2.49093 -0.82337 2.47284
H 3.34827 0.70996 2.43728
C -3.09479 1.65666 -0.33930
C -1.96279 2.69485 -0.24105
H -1.53329 2.75594 0.75721
H -2.37092 3.67821 -0.48852
H -1.16603 2.47563 -0.94825
C -4.26748 2.15300 0.51698
H -5.09679 1.45149 0.55317
H -4.64077 3.08672 0.08795
H -3.95677 2.36802 1.53916
C -3.51440 1.58329 -1.81357
H -2.70696 1.19485 -2.43284
H -3.74846 2.59417 -2.15829
H -4.40207 0.97365 -1.96650
C -3.57872 -1.42388 -0.23614
C -3.23926 -2.64447 0.63577
H -2.16966 -2.85143 0.62941
H -3.76234 -3.51199 0.22521
H -3.57745 -2.51773 1.66387
C -5.06476 -1.10226 -0.04557
H -5.28613 -0.80864 0.98128
H -5.64715 -2.00212 -0.26249
H -5.41622 -0.31862 -0.71279
C -3.29100 -1.79829 -1.69811
H -3.51560 -0.99030 -2.38966
H -3.91145 -2.65573 -1.97174
H -2.24504 -2.07306 -1.82417
C 3.05077 1.65718 -0.44014
C 1.91816 2.69630 -0.35186
H 1.09818 2.44549 -1.02022
H 2.31584 3.66759 -0.65774
H 1.52521 2.80316 0.65775
C 3.41919 1.50981 -1.92287
H 4.30275 0.89470 -2.07710
H 3.63763 2.50193 -2.32749
H 2.59140 1.08908 -2.49201
C 4.24594 2.20611 0.35068
H 3.96699 2.46621 1.37158
H 4.59003 3.12343 -0.13464
H 5.08700 1.52025 0.39128
C 3.61173 -1.39252 -0.15991
C 3.27309 -1.90987 -1.56625
H 2.24275 -2.25843 -1.61700
H 3.93520 -2.74498 -1.80900
H 3.40372 -1.14601 -2.32960
C 5.09407 -1.00939 -0.08462
H 5.38227 -0.28153 -0.83854
H 5.69385 -1.90835 -0.25293
H 5.36045 -0.61806 0.89740
C 3.39352 -2.53418 0.84850
H 3.84266 -2.30808 1.81486
H 3.87564 -3.43550 0.46334
H 2.34304 -2.76235 1.01388
Cl -0.02863 0.30178 -2.39395
N -0.03413 -2.13903 0.01315
H 0.68166 -2.84327 0.18811

(PNP)Re(NH_2)(Cl) (**3**):

```
Re -0.00000 -0.23772 -0.05234
Cl -0.00001 0.78018 -2.26960
P -2.38311 -0.04630 0.23011
P 2.38311 -0.04630 0.23011
N 0.00000 0.33599 1.81362
N -0.00000 -2.15104 -0.30770
H -0.82404 -2.73260 -0.34522
H 0.82403 -2.73260 -0.34521
C -2.45769 -0.00397 2.07428
H -3.35315 0.47212 2.46967
H -2.46553 -1.04071 2.40292
C -1.19800 0.65743 2.61152
H -1.32423 1.74577 2.66547
H -1.03615 0.32431 3.64262
C 1.19800 0.65742 2.61152
H 1.03615 0.32431 3.64262
H 1.32424 1.74577 2.66547
C 2.45769 -0.00398 2.07427
H 2.46553 -1.04072 2.40292
H 3.35315 0.47211 2.46967
C -3.17075 1.61358 -0.29375
C -2.11857 2.71934 -0.09270
H -1.75964 2.76736 0.93443
H -2.58004 3.68321 -0.32517
H -1.26613 2.58794 -0.75342
C -4.40340 2.01322 0.52925
H -5.20118 1.27813 0.49942
H -4.80125 2.94823 0.12444
H -4.14826 2.19859 1.57246
C -3.53010 1.57959 -1.78553
H -2.68013 1.26177 -2.38742
H -3.81144 2.58749 -2.10429
H -4.37451 0.92617 -1.99523
C -3.56387 -1.46421 -0.24773
C -3.26861 -2.66555 0.67182
H -2.20851 -2.82307 0.85870
H -3.67015 -3.57119 0.21099
H -3.75330 -2.54879 1.64076
C -5.06303 -1.17362 -0.11803
H -5.32825 -0.86045 0.89184
H -5.61704 -2.09274 -0.33184
H -5.40841 -0.41754 -0.81892
C -3.24901 -1.85543 -1.70077
H -3.46320 -1.04599 -2.39561
H -3.86465 -2.71287 -1.98551
H -2.20260 -2.12713 -1.82932
C 3.17076 1.61357 -0.29374
C 2.11859 2.71935 -0.09270
H 1.26616 2.58797 -0.75344
H 2.58008 3.68322 -0.32514
H 1.75964 2.76736 0.93442
C 3.53012 1.57959 -1.78552
H 4.37453 0.92616 -1.99521
H 3.81147 2.58749 -2.10428
H 2.68016 1.26178 -2.38742
C 4.40340 2.01320 0.52927
H 4.14827 2.19858 1.57247
H 4.80126 2.94822 0.12446
H 5.20118 1.27811 0.49943
C 3.56386 -1.46421 -0.24773
```

```
C 3.24900 -1.85543 -1.70077
H 2.20260 -2.12713 -1.82932
H 3.86464 -2.71288 -1.98551
H 3.46320 -1.04599 -2.39562
C 5.06302 -1.17362 -0.11804
H 5.40841 -0.41755 -0.81893
H 5.61703 -2.09274 -0.33185
H 5.32824 -0.86046 0.89184
C 3.26860 -2.66555 0.67181
H 3.75329 -2.54879 1.64076
H 3.67014 -3.57120 0.21098
H 2.20850 -2.82307 0.85869
```

(PNP)Re(NH_2)(Cl)$^+$ (**3**$^+$):

```
Re -0.00144 -0.21888 -0.11873
Cl 0.13092 0.88002 -2.19529
P -2.45508 0.05806 0.22269
P 2.44661 -0.09926 0.26375
N 0.00043 -0.15612 1.82287
N -0.04027 -2.04005 -0.67105
H -0.87898 -2.58825 -0.79954
H 0.77714 -2.61892 -0.79779
C -2.45293 0.30237 2.04211
H -2.45381 1.37683 2.21093
H -3.35240 -0.09770 2.50757
C -1.20582 -0.29282 2.66767
H -1.01849 0.20727 3.62074
H -1.34706 -1.35487 2.88987
C 1.18689 -0.21892 2.70197
H 0.97008 -0.91562 3.51556
H 1.33116 0.76059 3.16616
C 2.45191 -0.68412 1.99653
H 2.44046 -1.77032 1.94325
H 3.34128 -0.39124 2.55088
C -3.18302 1.68698 -0.40999
C -2.12362 2.78168 -0.18790
H -1.79691 2.84580 0.85031
H -2.56712 3.74452 -0.44998
H -1.24982 2.63425 -0.81580
C -4.45122 2.09939 0.35421
H -5.27542 1.40858 0.21140
H -4.76979 3.07437 -0.02186
H -4.27595 2.20810 1.42379
C -3.49498 1.59050 -1.90769
H -2.64244 1.22576 -2.47856
H -3.74579 2.58669 -2.27912
H -4.35037 0.94622 -2.10250
C -3.58003 -1.40785 -0.13251
C -3.11898 -2.53839 0.80646
H -2.04421 -2.70662 0.78621
H -3.61095 -3.46546 0.50633
H -3.40176 -2.33589 1.83910
C -5.06793 -1.15711 0.13396
H -5.25106 -0.77438 1.13788
H -5.60016 -2.10719 0.04208
H -5.50359 -0.47028 -0.58801
C -3.40128 -1.83054 -1.60009
H -3.85880 -1.11642 -2.28075
H -3.88834 -2.79541 -1.75513
H -2.35725 -1.92753 -1.89189
C 3.10627 1.68321 0.30153
C 2.01464 2.61701 0.85939
```

H 1.17965 2.73892 0.17244
H 2.45868 3.60453 1.00187
H 1.62872 2.29165 1.82280
C 3.44477 2.16356 -1.11637
H 4.30072 1.64748 -1.54381
H 3.69469 3.22596 -1.06894
H 2.59719 2.05057 -1.79029
C 4.33347 1.81808 1.21567
H 4.08463 1.61688 2.25714
H 4.68782 2.84994 1.16134
H 5.15747 1.17272 0.92930
C 3.61157 -1.23537 -0.70287
C 3.21762 -1.15239 -2.18621
H 2.18835 -1.46698 -2.35065
H 3.87221 -1.81010 -2.76177
H 3.32192 -0.14523 -2.58402
C 5.10203 -0.90960 -0.54483
H 5.37154 0.05803 -0.95823
H 5.67319 -1.66608 -1.08855
H 5.41798 -0.94795 0.49709
C 3.42081 -2.67951 -0.20720
H 3.90168 -2.83783 0.75706
H 3.88992 -3.35364 -0.92595
H 2.38111 -2.98185 -0.10538

Crystallographic details

Crystals were isolated in an Ar glovebox by decanting the mother liquor from the crystals before transferring and submerging them in high-viscosity petroleum oil on a microscope slide. Single crystals suitable for X-ray diffraction were identified under a polarizing microscope and mounted on 200 μm MiTeGen Dual-Thickness MicroLoops in a mixture of high-viscosity petroleum, then mounted on the diffractometer instrument. Low-temperature diffraction data (ω-scans) were collected on a Rigaku MicroMax-007HF diffractometer coupled to a Dectris Pilatus3R detector with Mo Kα (λ = 0.71073 Å) for the structure of 007c-17049 (**3**). Low-temperature diffraction data (ω-scans) were collected on a Rigaku MicroMax-007HF diffractometer coupled to a Saturn994+ CCD detector with Cu Kα (λ = 1.54178 Å) for the structure of 007b-17124 (**4**). The diffraction images were processed and scaled using either Rigaku CrystalClear software or Rigaku Oxford Diffraction software. The structure was solved with SHELXT and was refined against F^2 on all data by full-matrix least squares with SHELXL.

(PNP)Re(NH$_2$)(Cl) (3). All non-hydrogen atoms were refined anisotropically. Hydrogen atoms were included in the model at geometrically calculated positions and refined using a riding model. The isotropic displacement parameters of all hydrogen atoms were fixed to 1.2 times the U value of the atoms to which they are linked (1.5 times for methyl groups). The N-H positions are semi-freely refined with N-H distance restrains of 0.860(2) Å. The H-Re distances of H2a and H2b were restrained to be similar. The N-H hydrogen atoms are also riding on N2.

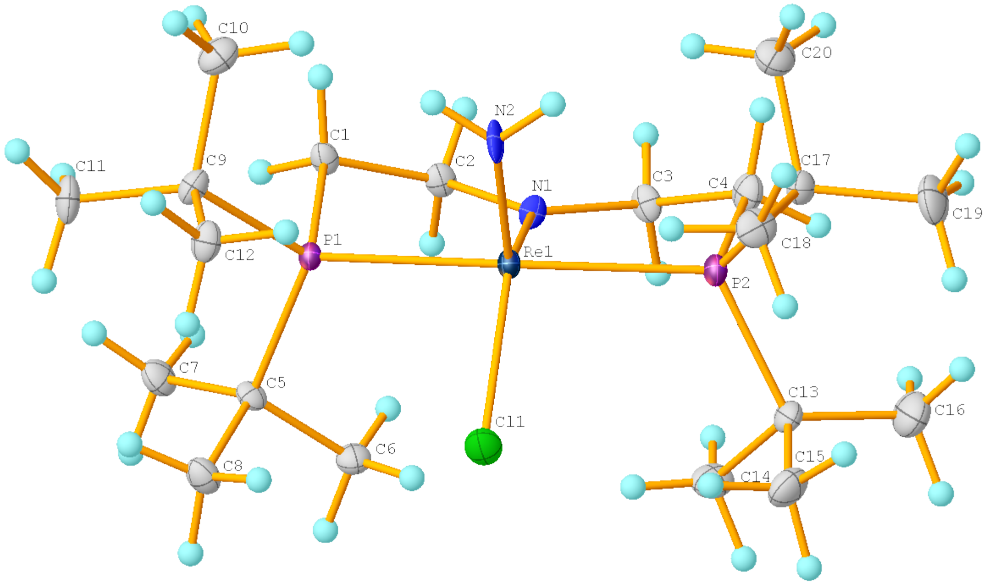

Figure S18. The complete numbering scheme of **3** with 50% thermal ellipsoid probability levels. The hydrogen atoms are shown as circles for clarity.

Table S10. Crystal data and structure refinement for **3**.

Identification code	007c-17049	
Empirical formula	C20 H46 Cl N2 P2 Re	
Formula weight	598.18	
Temperature	93(2) K	
Wavelength	0.71073 Å	
Crystal system	Monoclinic	
Space group	$P2_1/n$	
Unit cell dimensions	a = 8.5852(3) Å	α= 90°.
	b = 23.5810(7) Å	β= 92.515(3)°.
	c = 12.2292(4) Å	γ = 90°.
Volume	2473.39(14) $Å^3$	
Z	4	
Density (calculated)	1.606 g/cm^3	
Absorption coefficient	5.158 mm^{-1}	
F(000)	1208	
Crystal size	0.200 x 0.200 x 0.050 mm^3	
Crystal color and habit	Green Plate	
Diffractometer	Dectris Pilatus 3R	
Theta range for data collection	2.937 to 27.483°.	
Index ranges	-9<=h<=11, -30<=k<=30, -15<=l<=15	
Reflections collected	35252	
Independent reflections	5656 [R(int) = 0.0853]	
Observed reflections (I > 2sigma(I))	4806	
Completeness to theta = 25.242°	99.9 %	
Absorption correction	Semi-empirical from equivalents	
Max. and min. transmission	1.00000 and 0.53287	
Solution method	SHELXT-2014/5 (Sheldrick, 2014)	
Refinement method	SHELXL-2014/7 (Sheldrick, 2014)	
Data / restraints / parameters	5656 / 4 / 253	
Goodness-of-fit on F^2	1.008	
Final R indices [I>2sigma(I)]	R1 = 0.0290, wR2 = 0.0621	
R indices (all data)	R1 = 0.0383, wR2 = 0.0647	
Largest diff. peak and hole	1.871 and -1.100 e.$Å^{-3}$	

[(PNP)Re(NH_3)$_2$(Cl)][BAr^F$_4$] (4). All non-hydrogen atoms were refined anisotropically. Hydrogen atoms were included in the model at geometrically calculated positions and refined using a riding model. The isotropic displacement parameters of all hydrogen atoms were fixed to 1.2 times the U value of the atoms to which they are linked (1.5 times for methyl groups; 1.2 for NH_3 groups). The pentane and BAr^F sites are disordered. Four of the CF_3 groups of the BAr^F are disordered; the atoms involved are {F1, F2, F3}, {F7, F8, F9}, {F13, F14, F15} {C19, C20, C21}. Each group was modeled over two positions with their site occupancies freely refined. The two sites are distinguished with the suffixes "A" and "B". The converged values of the groups are 0.62(3):0.38(3), 0.60(3):0.40(3), 0.51(2):0.49(2), 0.613(12):0.378(12), respectively. The CF3 groups were restrained to have similar 1,2 C-C and C-F distances for chemically identical bonds. The 1,3 F-F distance were also restrained to be similar. The thermal parameters of the CF_3 groups were retained to behave as a rigid group. The pentane was disordered across the crystallographic inversion center. The special position constraints were suppressed and the site occupancies were fixed at 0.5. The pentane was retained to behave as a rigid group. The thermal parameter of C3A was restrained to be identical to that of its neighbour due to its proximity to the crystallographic inversion center.

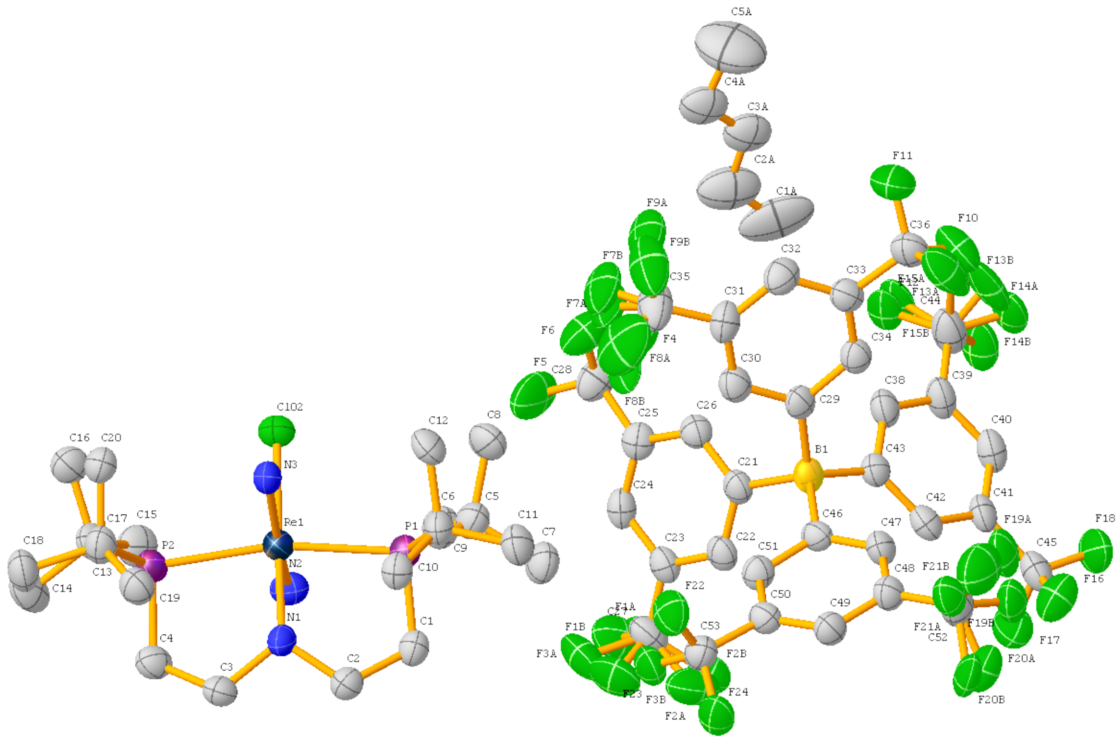

Figure S19. The complete numbering scheme of **4** with 50% thermal ellipsoid probability levels. The hydrogen atoms are omitted for clarity.

Table S11. Crystal data and structure refinement for **4**.

Identification code	007b-17124	
Empirical formula	C54.50 H68 B Cl F24 N3 P2 Re	
Formula weight	1515.51	
Temperature	93(2) K	
Wavelength	1.54184 Å	
Crystal system	Triclinic	
Space group	P-1	
Unit cell dimensions	a = 12.2681(5) Å	α= 92.777(3)°.
	b = 15.3208(6) Å	β= 109.357(4)°.
	c = 17.9247(8) Å	γ = 97.022(3)°.
Volume	3140.5(2) Å^3	
Z	2	
Density (calculated)	1.603 g/cm^3	
Absorption coefficient	5.646 mm^{-1}	
F(000)	1518	
Crystal size	0.050 x 0.050 x 0.010 mm^3	
Crystal color and habit	Green Plate	
Diffractometer	Rigaku Saturn 944+ CCD	
Theta range for data collection	2.625 to 66.597°.	
Index ranges	-14<=h<=14, -18<=k<=18, -21<=l<=21	
Reflections collected	109426	
Independent reflections	10946 [R(int) = 0.1340]	
Observed reflections (I > 2sigma(I))	8233	
Completeness to theta = 66.597°	98.4 %	
Absorption correction	Semi-empirical from equivalents	
Max. and min. transmission	1.00000 and 0.88256	
Solution method	SHELXT-2014/5 (Sheldrick, 2014)	
Refinement method	SHELXL-2014/7 (Sheldrick, 2014)	
Data / restraints / parameters	10946 / 295 / 922	
Goodness-of-fit on F^2	1.038	
Final R indices [I>2sigma(I)]	R1 = 0.0557, wR2 = 0.1212	
R indices (all data)	R1 = 0.0833, wR2 = 0.1364	
Largest diff. peak and hole	0.885 and -1.957 e.Å^{-3}	

References

1. García-Rodríguez, R.; Liu, H., *J. Am. Chem. Soc.* **2012,** *134*, 1400.
2. Manner, V. W.; Markle, T. F.; Freudenthal, J. H.; Roth, J. P.; Mayer, J. M., *Chem. Commun.* **2008**, 256-258.
3. Wise, C. F.; Agarwal, R. G.; Mayer, J. M., *J. Am. Chem. Soc.* **2020,** 142, 10681-10691.
4. Connor, G. P.; Mercado, B. Q.; Lant, H. M. C.; Mayer, J. M.; Holland, P. L., *Inorg. Chem.* **2019,** 58, 10791-10801.
5. Fulmer, G. R.; Miller, A. J. M.; Sherden, N. H.; Gottlieb, H. E.; Nudelman, A.; Stoltz, B. M.; Bercaw, J. E.; Goldberg, K. I., *Organometallics* **2010,** 29, 2176-2179.
6. Brunner, E., *Journal of Chemical & Engineering Data* **1985,** *30*, 269-273.
7. Frisch, M. J.; Trucks, G. W.; Schlegel, H. B.; Scuseria, G. E.; Robb, M. A.; Cheeseman, J. R.; Scalmani, G.; Barone, V.; Mennucci, B.; Petersson, G. A.; Nakatsuji, H.; Caricato, M.; Li, X.; Hratchian, H. P.; Izmaylov, A. F.; Bloino, J.; Zheng, G.; Sonnenberg, J. L.; Hada, M.; Ehara, M.; Toyota, K.; Fukuda, R.; Hasegawa, J.; Ishida, M.; Nakajima, T.; Honda, Y.; Kitao, O.; Nakai, H.; Vreven, T.; Montgomery Jr., J. A.; Peralta, J. E.; Ogliaro, F.; Bearpark, M. J.; Heyd, J.; Brothers, E. N.; Kudin, K. N.; Staroverov, V. N.; Kobayashi, R.; Normand, J.; Raghavachari, K.; Rendell, A. P.; Burant, J. C.; Iyengar, S. S.; Tomasi, J.; Cossi, M.; Rega, N.; Millam, N. J.; Klene, M.; Knox, J. E.; Cross, J. B.; Bakken, V.; Adamo, C.; Jaramillo, J.; Gomperts, R.; Stratmann, R. E.; Yazyev, O.; Austin, A. J.; Cammi, R.; Pomelli, C.; Ochterski, J. W.; Martin, R. L.; Morokuma, K.; Zakrzewski, V. G.; Voth, G. A.; Salvador, P.; Dannenberg, J. J.; Dapprich, S.; Daniels, A. D.; Farkas, Ö.; Foresman, J. B.; Ortiz, J. V.; Cioslowski, J.; Fox, D. J. *Gaussian 09*, Gaussian, Inc.: Wallingford, CT, USA, 2009.
8. Lindley, B. M.; van Alten, R. S.; Finger, M.; Schendzielorz, F.; Würtele, C.; Miller, A. J. M.; Siewert, I.; Schneider, S. *J. Am. Chem. Soc.* **2018**, *140*, 7922–7935.
